# Acupuncture-adjuvant therapies for treating perimenopausal depression: A network meta-analysis

**DOI:** 10.1097/MD.0000000000034694

**Published:** 2023-08-18

**Authors:** Lifang Zheng, Zhanling Sun, Chenghao Liu, Jiamin Zhang, Yabei Jin, Huifang Jin

**Affiliations:** a Zhejiang Hospital of Integrated Traditional Chinese and Western Medicine, Hangzhou, China; b Zhejiang Chinese Medical University, Hangzhou, China.

**Keywords:** acupuncture, depression, depression symptoms, menopause, network meta-analysis, perimenopause

## Abstract

**Background:**

The issues related to the treatment of perimenopausal depression (PMD) are the side effects of antidepressants and hormone replacement therapy. The aim of this study was to assess the efficiency and safety of acupuncture and moxibustion in PMD patients.

**Methods:**

Databases, namely PubMed, Cochrane Library, Web of Science, EMBASE, CNKI, CBM, VIP, and WanFang, were reviewed for related randomized controlled trials dated between database inception and November 22, 2022. The primary outcomes were the efficacy rate and the Hamilton Depression Scale score. The secondary outcomes were the levels of follicle-stimulating hormone, luteinizing hormone, and estradiol and the Kupperman score. Odds ratios (ORs) were generated as the effect size for dichotomous outcomes, while the standard mean difference (SMD) ± standard deviation was used for continuous outcomes. Matrices were developed to demonstrate pairwise comparisons of regimens related to each endpoint. Utilizing Review Manager (RevMan) 5.3, Stata 16.0 and SPSS 21, data were analyzed.

**Results:**

In total, 27 studies involving 2269 PMD patients and 8 therapeutic measures were incorporated into the network meta-analysis (NMA). The NMA showed that warm acupuncture (OR = 1.55, 95% CI: 1.00–2.44), electroacupuncture (OR = 1.34, 95% CI: 1.00–1.8), abdominal acupuncture (OR = 1.19, 95% CI: 0.73–1.96), and common acupuncture (OR = 1.4, 95% CI: 0.9–2.17) were more effective than fluoxetine + menopausal hormone treatment in the treatment of PMD. The NMA also showed that, based on the Hamilton Depression Scale score, warm acupuncture was more effective than the other 4 acupuncture-related treatments, i.e., electroacupuncture (SMD = −1.22, 95% CI: −2.34 to −0.09), thread embedding (SMD = −1.31, 95% CI: −2.21 to −0.40), abdominal acupuncture (SMD = −1.33, 95% CI: −2.42 to −0.24), and common acupuncture (SMD = −1.46, 95% CI: −2.26 to −0.66). The cumulative ranking probability (SUCRA) showed that warm acupuncture (99.6%) was the best treatment method.

**Conclusions:**

The findings of this network meta-analysis may help patients and therapists choose the best acupuncture therapy for treating perimenopausal depression patients and furnish reliable evidence for guidelines.

## 1. Introduction

With the increase in life expectancy, women now spend more than one-third of their lives in perimenopause or postmenopause.^[1]^ The perimenopausal period is a fragile window for the development of depressive symptoms and major depressive episodes.^[2]^ This period is characterized by dynamic changes in estradiol (E2), frequent stressful life events, and an increased risk of major depression.^[3]^ These events can also be accompanied by menopausal symptoms (such as vasomotor symptoms, insomnia, irritability, amnesia, and vaginal dryness).^[4]^ According to a study conducted in Taiwan, the incidence of depressive symptoms was 38.7%.^[5]^ Wang et al investigated perimenopausal women in 3 communities in Shanghai and showed that the incidence of depressive symptoms was 25.99%.^[6]^ Recent statistics showed that 53.1% of depressed patients in China have suicidal thoughts, which has a significant negative impact on families and society.^[7,8]^

Regarding the current treatment of perimenopausal depression (PMD), the 2018 guidelines recommend selective serotonin reuptake inhibitors and serotonin and norepinephrine reuptake inhibitors as PMD first-line drugs.^[9]^ Although antidepressants combined with menopausal hormone treatment (MHT) are recommended for most patients with menopausal depression, it has been observed that 70% of patients do not achieve complete recovery, and 30% do not respond to the first-line medication treatment.^[10]^ Additionally, studies have linked the use of hypnotic and anxiolytic drugs with a significantly increased risk of mortality.^[11]^ The side effects of those agents, such as dizziness, tremor, fatigue, mental damage, sexual problems, and urinary retention,^[12]^ which lead to reduced patient compliance, should not be neglected.^[13,14]^ In addition, although the Women’s Health Initiative (WHI) study corroborated the 5-year safety of estrogen + progesterone in breast and endometrial tissue, the long-term safety of hormone replacement therapy has yet to be proven.^[15–17]^

Therefore, people hope to find effective but less dangerous alternatives to achieve the effects of treatment and prevention effects. In this case, natural products, especially those from plants, that can effectively inhibit the central nervous system and alleviate related depressive symptoms have attracted attention as alternative drugs.^[18–22]^ Acupuncture and moxibustion, which are green and natural treatment methods in traditional Chinese medicine, are being welcomed by an increasing number of patients with depression because of their remarkable efficacy and lack of noticeable side effects. Moreover, traditional Chinese medical practices like acupuncture and moxibustion have demonstrated effectiveness in multiple studies as an additional or alternative treatment for PMD.^[23–25]^ Previous research by our team^[26,27]^ demonstrated the clinical efficacy and safety of acupoint catgut embedding in treating PMD. Our research group recently performed animal experiments. The results showed that acupuncture and moxibustion could regulate the behavioral symptoms of PMD rats and improve the levels of P13K, Akt, and mTOR in vivo.

There are many methods for acupuncture and moxibustion to treat PMD. Although previously published meta-analysis articles compared the therapeutic efficacy of acupuncture and medical therapies for PMD, few studies have been conducted on different methods of acupuncture and moxibustion for PMD. Thus, we considered the need for the current study in order to address these issues. This study adopts the mesh meta-analysis method to comprehensively analyze and evaluate relevant evidence. More studies have been included, as well as a broader population and an increased sample size, to lessen the random error, narrow the 95% confidence interval, and improve the stability of the results. This was done by carefully comparing a larger number of registered articles than that used in the other research. We aimed to explore the best acupuncture treatment for PMD and make it available to more people.

## 2. Materials and methods

### 2.1. Registration

This meta-analysis has been registered on PROSPERO, registration number CRD42022295453.

### 2.2. Literature search

**Figure 1.**
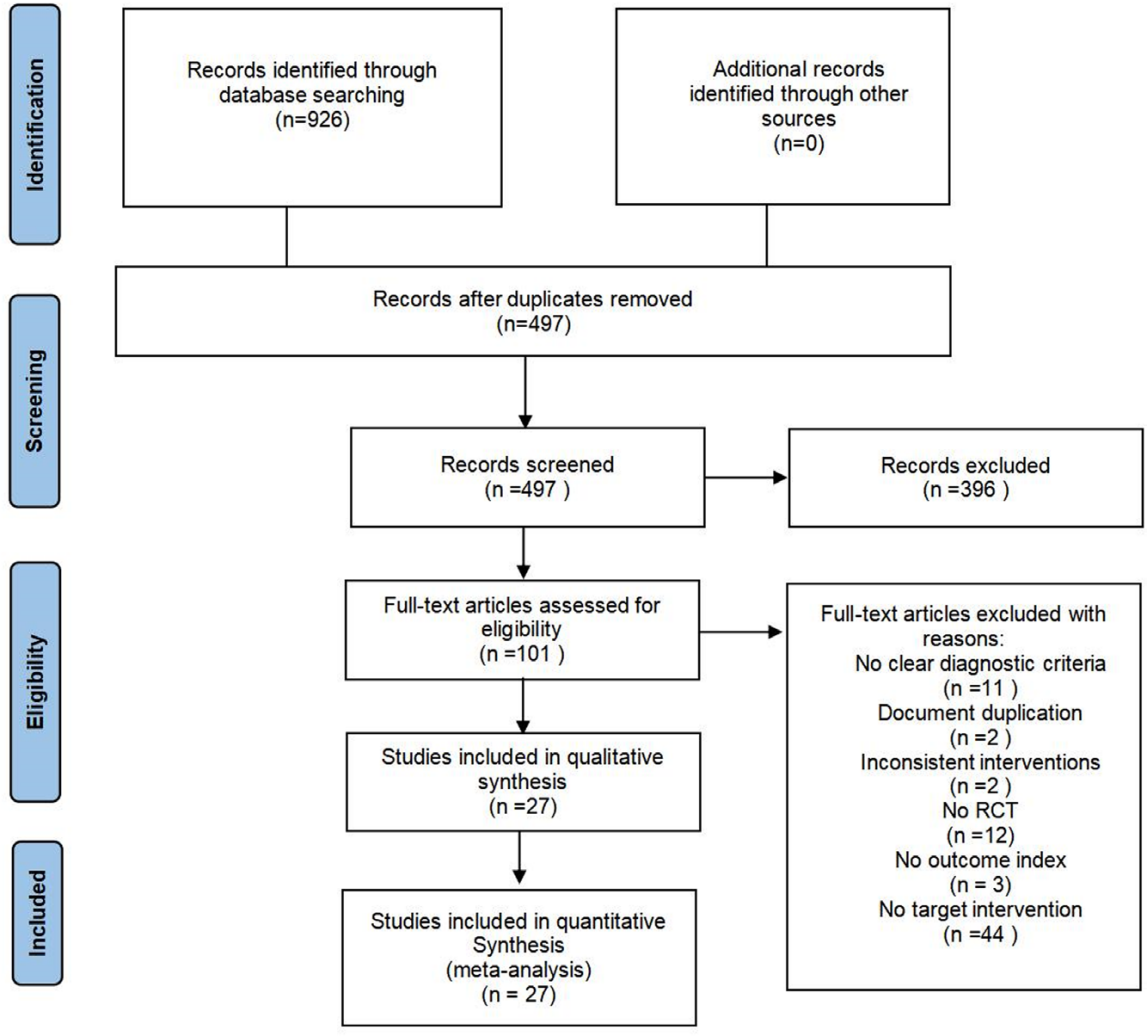
The PRISMA flow diagram of studies’ screening and selection.

Databases, namely PubMed, Cochrane Library, Web of Science, EMBASE, CNKI, CBM, VIP, and WanFang, were reviewed for related randomized controlled trials investigating the efficacy and safety of certain interventions for PMD between the time of database inception and November 22, 2022. The literature search was conducted using the following English terms: acupuncture, Pharmacopuncture, electropuncture, depression, depression symptom, menopause, perimenopause, and randomized controlled trials. The references cited by the relevant studies were searched manually. The Medical Subject Headings (MeSH) terms are detailed in the Table S1, Supplemental Digital Content, http://links.lww.com/MD/J460. The literature search process is shown in Figure 1.

### 2.3. Study selection

The inclusion criteria were as follows: Study design – Clinical randomized controlled trials of acupuncture for treating PMD were assessed. Intervention measures – The baseline treatment was consistent, and patients in the observation group were treated with only acupuncture and acupuncture-related therapies. Patients in the control group were treated with Western medicine, placebo, or ordinary acupuncture. (3) Study subjects – The included subjects met the diagnostic criteria for perimenopausal women and depression. The perimenopausal period was determined according to the standard proposed by the North American Menopause Association in 2012.^[28]^ The diagnosis of depression satisfied the criteria from at least one of the following: the International Classification of Diseases and Related Health Problems (ICD-10), Chapter 5, Mental Disorder Classification and Diagnostic Criteria^[29]^; the American Mental Disorder Classification System (DSM)^[30]^; and the third edition of the Chinese Classification and Diagnostic Standards for Mental Disorders (CCMD).^[31]^ The diagnosis was not subject to race, sex, occupation, disease course, TCM syndrome type, or other restrictions. The outcome measures were as follows: total effective rate; Hamilton Depression Scale (HAMD) score; KMI score; and levels of serum sex hormones such as follicle-stimulating hormone (FSH), luteinizing hormone (LH), and E2.

The exclusion criteria were as follows: Articles involving abstracts, conference papers, acupoint papers, and animal experiments were excluded. Acupuncture combined with other treatment methods was also rejected in the intervention group, such as acupuncture combined with Western medicine, acupuncture combined with traditional Chinese medicine, and acupuncture combined with ear point pressing beans. Duplicate publications and articles with unclear diagnostic or therapeutic evaluation criteria were also excluded.

### 2.4. Data extraction

Data from the studies were searched for, evaluated, and gathered independently by 2 researchers. A senior investigator adjudicated any discrepancy. The acquired data included the author’s name, country, publication year, sex, age, sample size, diagnostic criteria, interventions, outcome measures, adverse events, treatment duration, and follow-up time.

### 2.5. Article quality evaluation

The quality of the included articles was evaluated by the risk-of-bias assessment tool in the Cochrane Reviewers Handbook 6.1.0 using Review Manager 5.3. The risk of bias was summaried on the following aspects: random sequence generation, allocation concealment, blinding of participants and personnel, blinding of outcome evaluation, incomplete outcome data, selective reporting, and other bias.

### 2.6. Statistical analysis

The odds ratio (OR) and 95% confidence intervals (95% CIs) were generated as effect sizes for binary variants, while standard mean differences (SMDs) and 95% CIs were generated for continuous variants. Statistical heterogeneity was set as *I*^2^ > 50% or *P* < .01 for the random effects model. Otherwise, the fixed effect model was used. Network meta-analyses were executed according to the frequentist framework in Stata software version 17.0 MP by the random effects model to compare the overall effective rate, the HAMD score, and the sex hormone indices (FSH, LH, E2). The surfaces under the cumulative ranking curves (SUCRAs) were generated. A higher value of SUCRA indicated a higher probability of being the best treatment; however, the net-league table, also called the “matrix” in algebra, was used to determine whether the size of the effect between any 2 pairs reached significance. To enhance the stability of the results, gross and loop inconsistencies between direct and indirect comparisons were assessed. Funnel plots were generated to detect minor sample effects. *P* < .05 was regarded as statistically significant. Kappa consistency tests were run on the number of articles finally included and excluded by both reviewers using SPSS (version 21). This was used to assess the consistency of the results of the literature that had been screened by 2 reviewers and was eventually included in the analysis.

## 3. Results

### 3.1. Article search and screening

According to the retrieval strategy, a total of 936 articles were retrieved, including 220 articles in CNKI, 231 articles in Wanfang, 121 articles in VIP, 137 articles in CBM, 46 articles in PubMed, 106 articles in EMbase, 28 articles in Web of Science, 37 articles in Cochrane Library. In total, 27 studies were included in the meta-analysis after being found eligible following many layers of screening. We conducted Kappa consistency testing on the consistency of literature evaluations by 2 reviewers. The findings revealed a *P* value of .000 < .05 and the kappa value of 0.903 > 0.75, showing that the methodological consistency between the 2 reviewers on the inclusion and exclusion of the literature was statistically significant and the agreement was strong.

### 3.2. Characteristics of the included studies

A total of 936 studies were retrieved, 429 duplicated studies were excluded, 899 studies failed to match the inclusion criteria, and finally, 27 studies^[26,27,32–56]^ with 269 patients were included. The literature retrieval flowchart is shown in Figure 1, and the basic features of the included literature are shown in Table 1. The involved PMD patients met the diagnostic criteria for perimenopause and depression, which were determined according to the standard proposed by the North American Menopause Association in 2012. All studies reported treatment duration, and 6 of them reported follow-up results. The treatment duration ranged from 20 to 84 days. Among the 27 studies, 5 acupuncture treatment methods were identified: common acupuncture, electroacupuncture, warm acupuncture, abdominal acupuncture, and thread embedding. The included articles were evaluated for quality using the risk-of-bias assessment tool provided by the Cochrane Handbook. The basic information on the included literature is shown in Table 1.

**Table 1:** Basic characteristics of the included studies.

Study	Year	Group/size	Age (year)	Acupuncture interventions	Acupuncture interventions	Prescription in the control group	Prescription in the control group (method)	Outcome measure tool	Follow-up	Adverse events
Sheng et al	2018	T:116; C:105	T:49.83 ± 3.1; C:49.93 ± 3.1	Electroacupuncture	3 times/wk, 30 min, 12 w	Escitalopram	10 mg/1 time/d, 12 wk	HAMD, FSH, LH, E_2_	24-week follow-up	NA
Chen et al	2010	T:30; C:30	T:51.8 ± 4.2; C48.9 ± 3.8	Common acupuncture	6 times/wk, 30 min, 6 w	Fluoxetine	20 mg/1 time/d, 6 wk	HAMD, HAMA	No follow-up	NA
Chi et al	2011	T:30; C:30	T:51.63 ± 1.72; C:51.43 ± 1.62	Common acupuncture	1 time/day, 50 min, 4 w	Fluoxetine	20 mg/1 time/d, 4 wk	HAMD	No follow-up	T/n = 0; C/N = 3
Ding et al	2007	T:39; C:39	T49.68 ± 3.90; C49.50 ± 3.51	Common acupuncture	6 times/wk, 30 min, 4 w	Fluoxetine	20 g/1 time/d, 4 wk	HAMD, KMI	No follow-up	NA
Dong et al	2015	T:30; C:30	T:55; C:53	Common acupuncture	1 time/day, 30 min, 30 d	Fluoxetine	2 mg/1 time/d, 15 d; fluoxetine 20–40 mg/1 time/d, 30 d	HAMD	No follow-up	NA
Han et al	2016	T:38; C:35	T:46.7 ± 2.6; C:45.8 ± 2.3	Abdominal acupuncture	1 time/day, 30 min, 20 d	Deanxit	1 tablet/1 time/d, 20 d	SDS	No follow-up	T/n = 1; C/N = 4
Jin et al	2013	T:46; C:46	T:47.85 ± 4.32; C:48.52 ± 4.20	Thread embedding	1 time/wk, 8 w	Common acupuncture	3 times/wk, 30 min, 8 w	HAMD, FSH, LH, E2	No follow-up	NA
Li et al	2020	T:30; C:30	T:43~55; C:45~57	Electroacupuncture	1 time/qod, 25 min, 6 w	Fluoxetine	20 mg/1 time/d, 6 wk	HAMD, HAMA	No follow-up	NA
Li et al	2007	T:30; C:30	T:51.8 ± 4.2; C:48.9 ± 3.8	Common acupuncture	6 times/wk, 30 min, 6 w	Fluoxetine	20 mg/1 time/d, 6 wk	FSH, E2	No follow-up	NA
Liu et al	2019	T:29; C:29	T:51.48 ± 7.63; C:50.36 ± 7.94	Warm acupuncture	1 time/qod, 30 min, 3w	Common acupuncture	1 time/qod, 30 min, 3 wk	HAMD	No follow-up	NA
Ma et al	2009	T:30; C:30	NA	Common acupuncture	5 times/wk, 30 min, 8 w	Fluoxetine	20 mg/time/d, 8 wk	HAMD	No follow-up	NA
Qian et al	2007	T:33; C:33	T:54; C:55	Common acupuncture	5 times/wk, 25 min, 6 w	Fluoxetine	20 mg/1 time/d, 8 wk	HAMD	No follow-up	T/n = 2; C/N = 9
Wei et al	2008	T:30; C:30	T:54.32 ± 3.29; C:54.0 ± 4.62	Common acupuncture	5 times/wk, 25 min, 6 w	Fluoxetine	20 mg/1 time/d, 8 wk	HAMD	No follow-up	NA
Qing et al	2019	T:65; C:65	T:51.23 ± 1.32; C:50.12 ± 1.57	Thread embedding	1 time/2 wk, 4 w	Fluoxetine	20 mg/1 time/d, 8 w	HAMD	4-week follow-up	NA
Shi et al	2018	T:30; C:30	T:48.70 ± 1.99; C:49.43 ± 1.87	Electroacupuncture	3 times/wk, 30 min, 12 w	Escitalopram	10 mg/1 time/d, 12 w	HAMD	16–24-week follow-up	NA
Sun et al	2015	T:21; C:21	T:50.29 ± 2.59; C:49.86 ± 3.83	Electroacupuncture	3 times/wk, 30 min, 12 w	Escitalopram	10 mg/1 time/d, 12 w	HAMD	No follow-up	NA
Sun et al	2015	T:22; C:22	T:45 ± 4; C:47 ± 4	Thread embedding	1 time/wk, 8 w	Acupuncture	1 time/qod/30 min; 8 w	HAMD, KMI	12-week follow-up	NA
Wang et al	2014	T:35; C:35; I:35	T: 48.72 ± 4.21; C:48.64 ± 4.82; S:47.82 ± 4.35	Common acupuncture	3 times/wk, 30 min, 8 w	Wuling capsule; sham acupuncture group	3 pills/1 time, 3 times/d, 8 w; shallow needling (without waiting for the acupuncture feeling)	SDS	No follow-up	NA
Wang et al	2018	T:30; C:30	T:50.1 ± 4.71; C:49.5 ± 4.32	Pressing acupuncture	1 time/day, 12 w	Deanxit	1 tablet 2 times/d, 1–4 weeks; 1 tablet 1 time/d, 4–8 w; 0.5 tablet 1 time/d, 8–12 w	SAS, SDS, KMI	No follow-up	T/n = 3; C/N = 4
Wang et al	2010	T:30; C:30	T:49.6 ± 4.3; C:48.3 ± 4.7	Abdominal acupuncture	1 time/day, 30 min, 3 days; 1 time/3 days, 4 w	Deanxit	1 tablet/1 time/d, 4 w	HAMD	2–4-week follow-up	T/n = 3; C/N = 15
Xiang et al	2013	T:23; C:23	T:49 ± 4; C:48 ± 3	Thread embedding	1 time/wk, 8 w	Acupuncture	1 time/qod, 30 min, 8 w	HAMD, KMI	No follow-up	NA
Xing et al	2011	T:120; C:120	T:51.2 ± 5.4; C:49.5 ± 6.8	Common acupuncture	1 time/day, 20 min, 6 w	Fluoxetine	20 mg/1 time/d, 6 w	HAMD	No follow-up	NA
Zhang et al	2013	T:94; C:94	T:50.1 ± 2.7; C:49.8 ± 2.6	Common acupuncture	1 time/day, 20 min, 3 months	Fluoxetine + MHT	20 mg/1 time/d, 6w + MHT	HAMD, FSH, LH, E2	No follow-up	T/n = 2; C/N = 5
Zheng et al	2018	T:40; C:40	T:50.78 ± 0.54; C:50.82 ± 0.58	Common acupuncture	5 times/wk, 20 min, 6 w	Fluoxetine	20 mg/1 time/d, 6 w	HAMD, FSH, LH, E2	No follow-up	NA
Zheng et al	2010	T:60; C:60	T:52.27 ± 3.45; C: 51.98 ± 3.14	Common acupuncture	1 time/day, 20 min, 3 months	Fluoxetine + MHT	20 mg/1 time/d, 6w + MHT	HAMD, KMI, FSH, LH, E2	6-month follow-up	T/n = 2; C/N = 5
Zhou et al	2007	T:30; C:30	T:49.6 ± 3.2; C:49.6 ± 3.2	Common acupuncture	6 times/wk, 30 min, 6 w	Fluoxetine	20 mg/1 time/d, 6 w	FSH, E2	No follow-up	NA
Zhou et al	2007	T:30; C:30	T:51.8 ± 4.2; C:48.9 ± 3.8	Common acupuncture	6 times/wk, 30 min, 6 w	Fluoxetine	20 mg/1 time/d, 6 w	HAMD	No follow-up	NA

C = control group, E2 = estradiol, FSH = follicle-stimulating hormone, HAMD = Hamilton Depression Scale, KMI = Kupperman index, LH = luteinizing hormone, MHT = menopausal hormone treatment, NA = not available, S = sham acupuncture group, SDS = Self-Rating Depression Scale, T = test group.

### 3.3. Risk of bias

**Figure 2.**
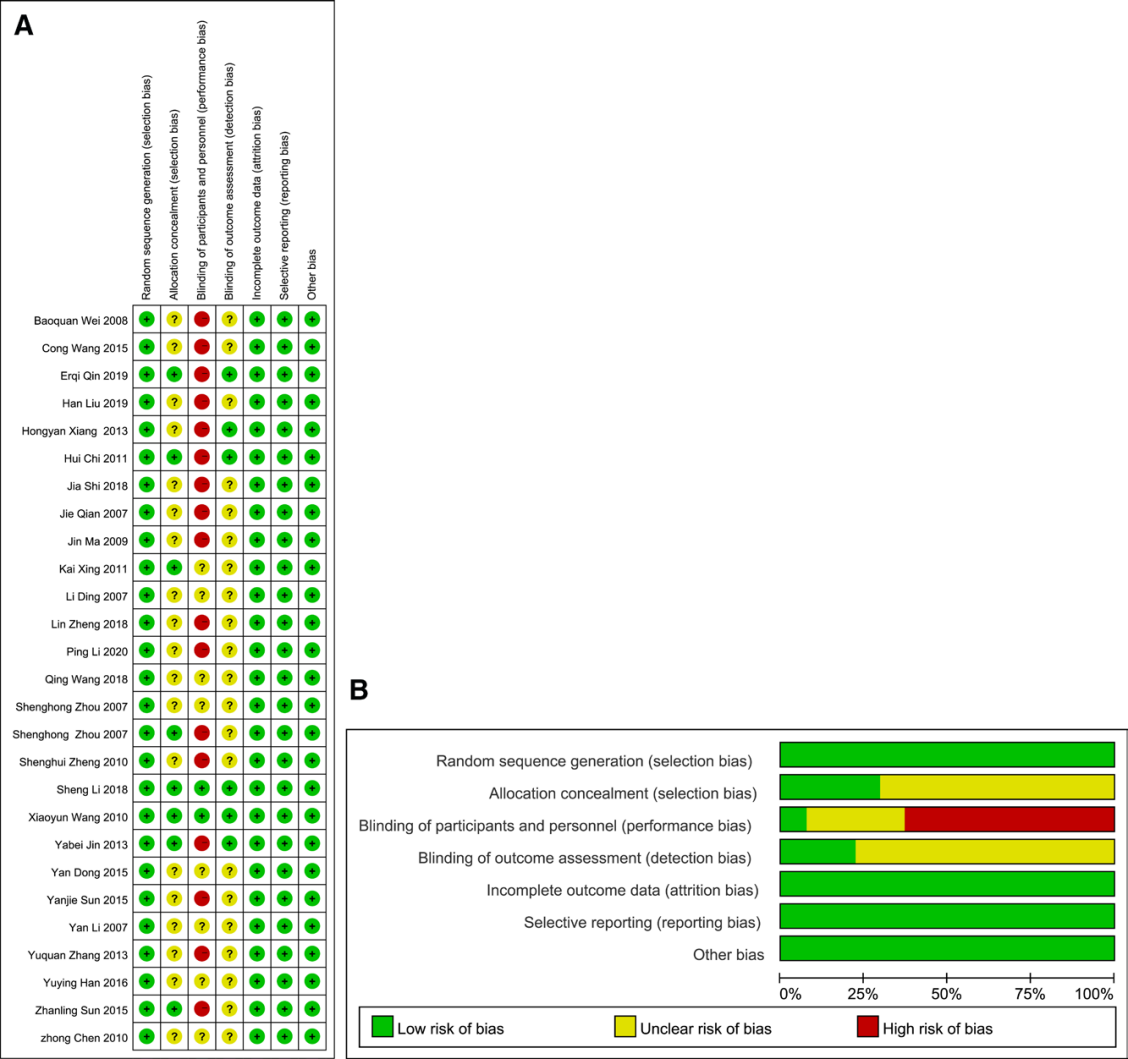
(A) Risk of bias summary; (B) Risk of bias graph.

Thirteen studies used the random number table method for random assignment,^[26,27,34–36,39,41,43,48,49,51]^ and one study used the lottery for random assignment.^[51]^ One study used the order of visits for assignment^[45]^ and was rated as “low risk.” The other 14 studies mentioned using random assignment but did not specify the specific method and were rated as “unclear risk.” Eight studies^[26,27,35,37,41,43,47,49]^ mentioned allocation concealment and described it as “low risk,” while the remaining exact studies did not mention allocation concealment and were rated as “unclear risk.” Six studies^[26,35–37,43,49]^ mentioned the implementation of blinding in the evaluation of the result and were rated as “low risk,” while the remaining 21 studies did not mention allocation concealment and were rated as “unclear risk.” One study^[43]^ mentioned that blinding was implemented as double-blinding and was rated as “low risk”; the remaining 26 studies did not report the use of blinding and were rated as “unclear risk.” All 27 studies reported expected measured outcome indicators. We did not identify any cases of early trial terminations. Incomplete data and selective reports were rated as “low risk.” None of the 27 studies described other biases in detail and were rated as “unclear risk.” The results of the risk assessment of the article bias are shown in Figure 2A and B.

### 3.4. Results of the evaluation of basic characteristics

For convenience, 15 of the 27 trials^[32,33,37,39–42,44,47,48,50,51,53,55,56]^ compared common acupuncture and fluoxetine, fluoxetine + MHT, and sham acupuncture; 4 trials^[38,43,45,52]^ compared electroacupuncture and fluoxetine or escitalopram; 4 trials^[26,27,34,36]^ compared thread embedding and common acupuncture; 2 trials^[49,54]^ compared abdominal needle against Deanxit; and 1 trial^[35]^ compared warm needle with common acupuncture and pressing needle acupuncture with Deanxit.^[46]^ The results of the literature quality evaluation are shown in Table 1.

### 3.5. Results of the network meta-analysis (NMA)

**Figure 3.**
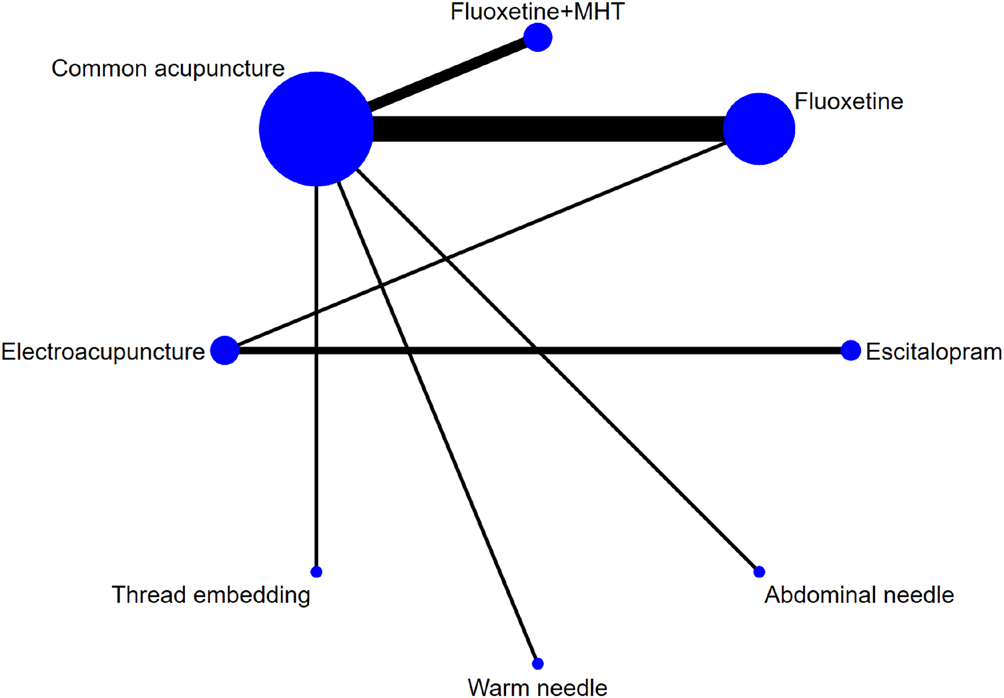
Evidence network diagram of efficacy rate. MHT = menopausal hormone treatment.

**Figure 4.**
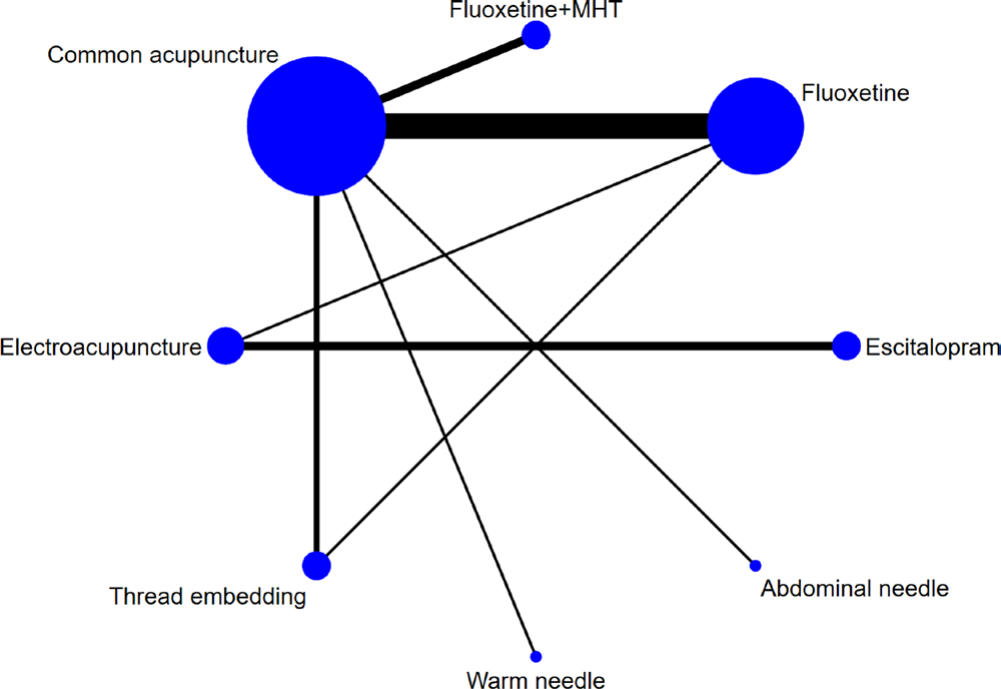
Evidence network diagram of HAMD scores. HAMD = Hamilton Depression Scale, MHT = menopausal hormone treatment.

The network plots of the effective rate and the HAMD score are presented in Figures 3 and 4. Each spot represents an intervention, and the area of the spot corresponds to the number of patients who received that particular intervention. The number of trials is represented by the width of the line between each 2 points.

### 3.6. Overall efficacy rate

**Figure 5.**
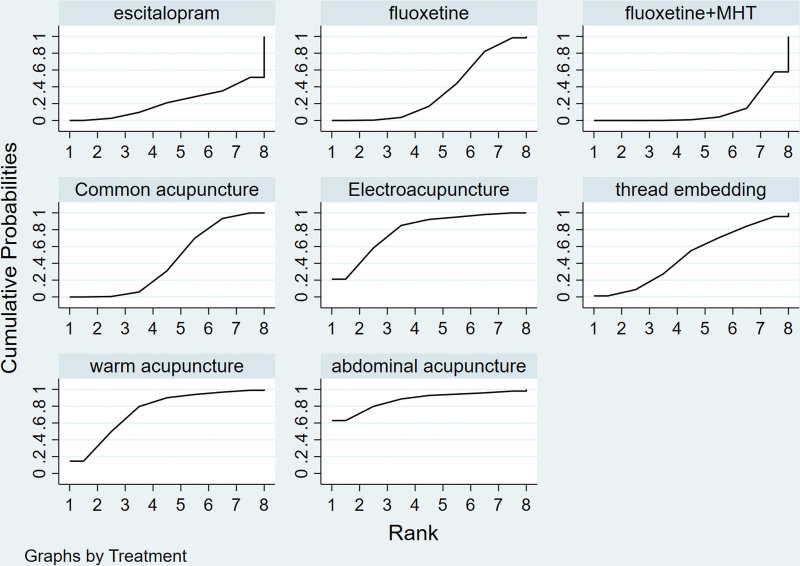
Efficiency SUCRA diagram. SUCRA = the cumulative ranking probability.

A total of 16 studies reporting on clinical effectiveness involved 8 treatment measures. The results of the NMA showed that, in terms of improving the total efficiency rate, warm acupuncture (OR = 1.55, 95% CI: 1.00–2.44), electroacupuncture (OR = 1.34, 95% CI: 1.00–1.8), abdominal acupuncture (OR = 1.19, 95% CI: 0.73–1.96), and common acupuncture (OR = 1.4, 95% CI: 0.9–2.17) had higher total efficacies than fluoxetine + MHT. Electroacupuncture (OR = 1.35, 95% CI: 1.09–1.67) was also superior to escitalopram (Table 2). Taking the total effective rate as the outcome index, the SUCRA method was used to rank the advantages and disadvantages of the intervention measures. The larger the area under the curve, the more effective the intervention. The SUCRA ranking results showed that the best treatment was abdominal acupuncture (SUCRA = 87.9%), followed by electroacupuncture (SUCRA = 78.6%), warm acupuncture (SUCRA = 74.2%), thread embedding (SUCRA = 48.5%), common acupuncture (SUCRA = 43.3%), fluoxetine (SUCRA = 35.1%), fluoxetine + MHT (SUCRA = 21.2%), and escitalopram (SUCRA = 11.1%). Additional supporting evidence from the results of the NMA revealed that warming acupuncture was the most efficacious intervention for PMD compared to the other 4 acupuncture-related treatments (Fig. 5).

**Table 2:** Network meta-analysis of total effectiveness.

Interventions	Warm acupuncture	Electroacupuncture	Abdominal acupuncture	Thread embedding	Common acupuncture	Fluoxetine	Escitalopram	Fluoxetine + MHT
Warm acupuncture	0	0.86 (0.51, 1.45)	0.84 (0.51, 1.38)	0.74 (0.46, 1.18)	0.71 (0.46, 1.11)	0.70 (0.45, 1.10)	0.64 (0.37, 1.12)	0.64 (0.41, 1.01)
Electroacupuncture	1.16 (0.69, 1.95)	0	0.97 (0.68, 1.39)	0.85 (0.61, 1.18)	0.83 (0.63, 1.09)	0.81 (0.62, 1.06)	0.74 (0.60, 0.92)	0.75 (0.56, 1.00)
Abdominal acupuncture	1.19 (0.73, 1.96)	1.03 (0.72, 1.47)	0	0.88 (0.65, 1.18)	0.85 (0.67, 1.08)	0.84 (0.66, 1.07)	0.76 (0.50, 1.16)	0.77 (0.60, 0.99)
Thread embedding	1.36 (0.85, 2.18)	1.17 (0.85, 1.63)	1.14 (0.85, 1.53)	0	0.97 (0.81, 1.16)	0.96 (0.79, 1.16)	0.87 (0.59, 1.29)	0.87 (0.71, 1.07)
Common acupuncture	1.40 (0.90, 2.17)	1.21 (0.92, 1.59)	1.17 (0.93, 1.48)	1.03 (0.86, 1.23)	0	0.98 (0.92, 1.05)	0.90 (0.63, 1.27)	0.90 (0.82, 0.99)
Fluoxetine	1.42 (0.91, 2.22)	1.23 (0.94, 1.60)	1.19 (0.94, 1.52)	1.05 (0.87, 1.27)	1.02 (0.95, 1.09)	0	0.91 (0.65, 1.28)	0.92 (0.81, 1.03)
Escitalopram	1.56 (0.89, 2.73)	**1.35 (1.09, 1.67**)	1.31 (0.86, 1.99)	1.15 (0.78, 1.70)	1.12 (0.79, 1.58)	1.10 (0.78, 1.54)	0	1.00 (0.70, 1.44)
Fluoxetine + MHT	**1.55 (1.00, 2.44**)	**1.34 (1.00, 1.80**)	**1.30 (1.01, 1.68**)	1.14 (0.93, 1.40)	**1.11 (1.01, 1.23**)	1.09 (0.97, 1.23)	1.00 (0.69, 1.43)	0

The difference between the 2 groups was statistically significant. Significant results are in bold: *P* < .05.

MHT = menopausal hormone treatment.

### 3.7. HAMD score results

**Figure 6.**
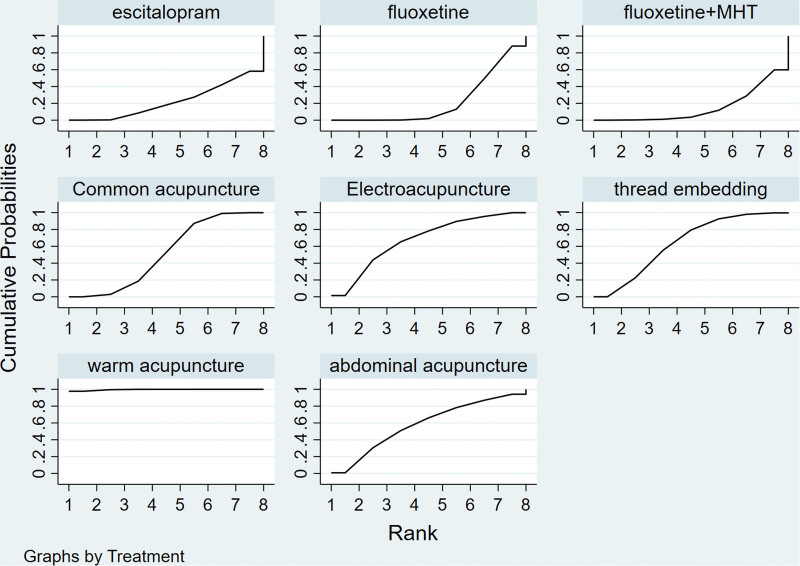
SUCRA chart of HAMD score. HAMD = Hamilton Depression Scale, SUCRA = the cumulative ranking probability.

Twenty-one of 27 studies reported the HAMD score of PMD symptoms. From the matrix (Table 3), among the 8 interventions, there were statistically significant differences between warm acupuncture and the other 7 treatments, i.e., electroacupuncture [SMD = −1.22, 95% CI (−2.34 to −0.09)], thread embedding [SMD = −1.31, 95% CI (−2.21 to −0.40)], abdominal acupuncture [SMD = 1.33, 95% CI (−2.42 to −0.24)], common acupuncture [SMD = −1.46, 95% CI (−2.26 to −0.66)], escitalopram [SMD = −1.81, 95% CI (−3.00 to −0.61)], fluoxetine [SMD = −1.74, 95% CI (−2.57 to −0.9)], and fluoxetine + MHT [SMD = −1.84, 95% CI (−2.73 to −0.95)]. The results showed that warm acupuncture was the best treatment for PMD. Compared with escitalopram, electroacupuncture [SMD = −0.59, 95% CI (−1.01 to −0.17)] markedly reduced HAMD scores. Compared to fluoxetine, ordinary acupuncture [SMD = −2.8, 95% CI (−0.51 to −0.05)] effectively reduced HAMD scores. According to the Sucra score, warming acupuncture had the highest ranking (SUCRA = 99.6%), followed by electroacupuncture (SUCRA = 67.7%), thread embedding (SUCRA = 64%), abdominal acupuncture (SUCRA = 58.2%), and common acupuncture (SUCRA = 51.5%). Additional supporting evidence from the SUCRA plot revealed that warming acupuncture had the maximum area under the curve. Hence, warming acupuncture was the most efficacious intervention for improving the HAMD score in PMD compared to the other 4 acupuncture-related treatments (Fig. 6).

**Table 3:** Network meta-analysis of HAMD score.

Interventions	Warm acupuncture	Electroacupuncture	Thread embedding	Abdominal acupuncture	Common acupuncture	Escitalopram	Fluoxetine	Fluoxetine + MHT
Warm acupuncture	0	1.22 (0.09, 2.34)	1.31 (0.40, 2.21)	1.33 (0.24, 2.42)	1.46 (0.66, 2.26)	1.81 (0.61, 3.00)	1.74 (0.90, 2.57)	1.84 (0.95, 2.73)
Electroacupuncture	**−1.22 (−2.34, −0.09**)	0	0.09 (−0.77, 0.96)	0.11 (−0.97, 1.20)	0.24 (−0.54, 1.03)	0.59 (0.17, 1.01)	0.52 (−0.23, 1.27)	0.62 (−0.25, 1.50)
Thread embedding	**−1.31 (−2.21, −0.40**)	−0.09 (−0.96, 0.77)	0	0.02 (−0.83, 0.88)	0.15 (−0.27, 0.57)	0.50 (−0.47, 1.46)	0.43 (−0.00, 0.86)	0.53 (−0.04, 1.10)
Abdominal acupuncture	**−1.33 (−2.42, −0.24**)	−0.11 (−1.20, 0.97)	−0.02 (−0.88, 0.83)	0	0.13 (−0.62, 0.87)	0.48 (−0.68, 1.64)	0.41 (−0.37, 1.19)	0.51 (−0.33, 1.35)
Common acupuncture	**−1.46 (−2.26, −0.66**)	−0.24 (−1.03, 0.54)	−0.15 (−0.57, 0.27)	−0.13 (−0.87, 0.62)	0	0.35 (−0.54, 1.24)	0.28 (0.05, 0.51)	0.38 (−0.01, 0.77)
Escitalopram	**−1.81 (−3.00, −0.61**)	**−0.59 (−1.01, −0.17**)	−0.50 (−1.46, 0.47)	−0.48 (−1.64, 0.68)	−0.35 (−1.24, 0.54)	0	−0.07 (−0.93, 0.79)	0.03 (−0.94, 1.00)
Fluoxetine	**−1.74 (−2.57, −0.90**)	−0.52 (−1.27, 0.23)	−0.43 (−0.86, 0.00)	−0.41 (−1.19, 0.37)	**−0.28 (−0.51, −0.05**)	0.07 (−0.79, 0.93)	0	0.10 (−0.35, 0.55)
Fluoxetine + MHT	**−1.84 (−2.73, −0.95**)	−0.62 (−1.50, 0.25)	−0.53 (−1.10, 0.04)	−0.51 (−1.35, 0.33)	−0.38 (−0.77, 0.01)	−0.03 (−1.00, 0.94)	−0.10 (−0.55, 0.35)	0

The difference between the 2 groups was statistically significant. Significant results are in bold: *P* < .05.

HAMD = Hamilton Depression Scale, MHT = menopausal hormone treatment.

### 3.8. Secondary results

The network plots of the KMI scores, FSH, LH, and E2 levels are presented in Figures S7–S10, Supplemental Digital Content, http://links.lww.com/MD/J461. Five studies reported KMI scores, 4 studies reported LH levels, and 6 studies reported FSH and E2 levels. Regarding improvement in E2 levels, the 4 treatment methods used were common acupuncture, thread embedding, fluoxetine, and fluoxetine + MHT. According to the matrix, fluoxetine + MHT [SMD = 1.6, 95% CI (0.57–2.64)] and common acupuncture [SMD = 0.85, 95% CI (0.18–1.51)] were significantly inferior to fluoxetine, and fluoxetine + MHT was significantly inferior to common acupuncture [SMD = 1.3, 95% CI (0.04–0.55)]. There was no statistically significant difference between the groups in KMI, FSH, or LH levels (see Tables S2–S5, Supplemental Digital Content, http://links.lww.com/MD/J462, which illustrated the above results. The sucra score graph of secondary results could be seen in the supplementary Figures S11–S14, http://links.lww.com/MD/J463 and The relevant description of the secondary indicators were explained in the Supplemental Digital Content, http://links.lww.com/MD/J465).

### 3.9. Publication bias analysis

Publication bias analysis of the overall efficacy rate, the HAMD score and the secondary results (the KMI score, LH, FSH, and E2 levels outcome can be seen in Figures S1–S6, Supplemental Digital Content, http://links.lww.com/MD/J464. The funnel chart shows that the inverted funnel plot is essentially symmetrical, it is possible that this study has a small sample effect or a small publication bias.

## 4. Discussion

To the best of our knowledge, this is the first network meta-analysis of various therapies in patients with PMD. As a natural therapy with a positive therapeutic effect, acupuncture has become a research hotspot in the treatment of PMD. According to the results of the current network meta-analysis and the ranking of intervention measures, we found that warm acupuncture had a prominent impact on the efficacy rate of improving PMD and HAMD scores compared to all other acupuncture-related therapies. This is slightly different from our traditional clinical experience, suggesting that electroacupuncture would be an effective method to improve PMD. Recent research shows that electroacupuncture at Baihui, Yintang, Tianshu, and the uterus can improve the clinical symptoms of PMD patients.^[57]^ Many studies have also shown that acupuncture and moxibustion could significantly improve the symptoms of PMD.^[58]^ Acupuncture and moxibustion can reduce perimenopausal symptoms by regulating the Hypothalamic Pituitary Axis and affecting monoamine neurotransmitters, their receptors, and their signaling pathways. The symptoms of PMD can also be alleviated by upregulating the expression of 5-HT,^[59]^ EAAT2,^[60]^ and PKA/CREB^[61]^ and regulating the secretion function of the hypothalamic–pituitary–ovarian axis.^[62]^

The significant effect of warm acupuncture on PMD is related to the unique theory of warm acupuncture, which is a combination of moxibustion and acupuncture. This method is performed by inserting a needle into a point and then burning a moxa stick on the needle’s handle.^[63]^ Heat is introduced into the acupoints through the needle’s body, which has both acupuncture and warming effects. According to the Huangdi Internal Classic of Chinese Medicine, “moxibustion is necessary when needles cannot reach.” Recent studies have shown that moxibustion includes not only the transformation of heat stimulus signals but also the transformation of light radiation energy signals.^[64]^ The light and heat stimulation of moxibustion can play a regulatory role in all systems of the body. It can stimulate the specific immune function of the body, helping to normalize immune function.^[65]^ Research shows that acupuncture combined with warming can regulate the overall condition of the body, promote local circulation, accelerate the metabolism of inflammatory substances,^[66]^ and effectively improve the symptoms of PMD.^[67–69]^ Mechanistically, this is consistent with modern research on PMD. Current research suggests that neurodegenerative diseases such as PMD may be closely related to neuroinflammatory cytokines, cerebral hemodynamics, and blood–brain barrier dysfunction in the body.^[70]^ In addition, studies have demonstrated that warm acupuncture can improve levels of thromboxane B2 (TXB2), tumor necrosis factor-α, and interleukin-6 (IL-6).^[71]^ It can also regulate immune factors, immune organs, and nerve conduction to treat diseases.^[72]^ Our recent animal experiments also revealed that acupuncture and moxibustion could regulate the behavioral symptoms of PMD rats and improve the levels of P13K, Akt, and mTOR in vivo. PMD symptoms may be related to autophagy of hippocampal kidney meridians.

Next, we can attempt to observe and discuss the possible mechanism of warm acupuncture in PMD patients according to the results of the network meta-analysis and in combination with the previous experimental basis. A standard for managing PMD can be established from the perspectives of the condition’s diagnosis, medicine, and traditional Chinese acupuncture treatment, which will promote the clinical use of warm acupuncture as a supplemental and alternative therapy for PMD.

## 5. Limitations

Although the current network meta-analysis strongly suggests that acupuncture-related treatment is more effective than Western medicine in improving symptoms of PMD, the present study still has some limitations.

First, the substance of some research was of poor quality. Randomized controlled trials in the literature usually do not have a strict study design, nor do they have a detailed description of randomization, allocation concealment, or blinding methods. Second, the number of documents that met the inclusion criteria was relatively small, and the sample size of some documents was insufficient. Finally, there were not enough acupuncture interventions that met the inclusion criteria, which affected the reliability of the evaluation results.

## 6. Conclusions

The current evidence shows that warm acupuncture is effective in improving the total efficacy rate and HAMD score in perimenopausal patients with depression. Due to the quality limitation of the study, a stricter design and more standardized reports are required to further demonstrate the reliability of the findings. In the future, it should be examined whether acupuncture and moxibustion combined with medications used for PMD are effective.

## Acknowledgments

We thank all the authors of the studies included and Prof Jin Yabei for her valuable comments on the article. We thank Dr Chen Xi from the Department of Epidemiology and Statistics, School of Medicine, Zhejiang University for guiding the statistical analysis of this article. We also thank LetPub (www.letpub.com) for its linguistic assistance during the preparation of this manuscript.

## Author contributions

**Data curation:** Lifang Zheng, Zhanling Sun, Chenghao Liu, Jiamin Zhang.

**Methodology:** Lifang Zheng, Zhanling Sun, Chenghao Liu.

**Project administration:** Huifang Jin.

**Resources:** Lifang Zheng.

**Supervision:** Yabei Jin.

**Writing – original draft:** Lifang Zheng.

**Writing – review & editing:** Lifang Zheng, Huifang Jin.

## Supplementary Material












