# Supplementary material for: Acupuncture-adjuvant therapies for treating perimenopausal depression: A network meta-analysis
Source: Medicine (Baltimore). 2023 Aug 18;102(33):e34694. doi: 10.1097/MD.0000000000034694 (PMC10443772; doi:10.1097/MD.0000000000034694)
Supplement: Supplementary file 1 [file medi-102-e34694-s001.pdf]

Table S1. Chinese and English search strategies

Comments:

In order for non-Chinese readers to understand the Chinese search strategy of this article, we translated the Chinese search terms in the search formula.

CNKI	(SU=Perimenopausal OR SU=Menopausal OR SU=Menopausal Syndrome OR SU=Perimenopausal Syndrome OR SU=Menopausal Transition Period) AND (SU=Depression OR SU=Anxiety OR SU=Abnormal Emotion OR SU=Depressive Symptoms OR SU=Depressive Mood) AND (SU=Acupuncture OR SU=Ear Acupuncture OR SU=Abdominal Acupuncture OR SU=Scalp Acupuncture OR SU=Body Acupuncture OR SU=Electroacupuncture OR SU=Plum-Blossom Acupuncture OR SU=Water Acupuncture OR SU =Floating Acupuncture OR SU=Warm Acupuncture OR SU=Umbilical Acupuncture OR SU=Thread Embedding OR SU=Acupuncture Therapy) AND (FT=Random or FT=Randomized Control or FT=Randomized or FT=rct or FT=RCT
WanFang Database	Subject: (Perimenopausal or Menopause or Menopausal Syndrome or Perimenopausal Syndrome or Menopausal Transition) and Subject: (Depression or Anxiety or Emotional Abnormalities or Depressive Symptoms or Depression) and Subject: (Acupuncture or Ear Acupuncture or Abdominal Acupuncture or Scalp Acupuncture or Body Acupuncture or Electrocupuncture or Plum Blossom Acupuncture or Water Acupuncture or Floating Acupuncture or Warm Acupuncture or Umbilical Acupuncture or Thread Embedding or Acupuncture Therapy) and all: (Randomized or Randomized Controlled or Random Grouping or rct or RCT)
CSJD-VIP	M=(Perimenopausal OR Menopause OR Menopausal Syndrome OR Perimenopausal Syndrome OR Menopausal Transition) AND M=(Depression OR Anxiety OR Emotional Abnormal OR Depressive Symptom) AND M=(Acupuncture OR Ear Acupuncture OR Abdominal Acupuncture OR Scalp Acupuncture OR Body Acupuncture OR Electroacupuncture OR Plum Blossom Acupuncture OR Water Acupuncture OR Floating Acupuncture OR Warm Acupuncture OR Umbilical Acupuncture OR Embedding OR Acupuncture Therapy) AND U=(Random OR Randomized Control OR Random Grouping OR rct OR RCT)
CBM	(Perimenopausal [Title] OR Menopause [Title] OR Menopausal Syndrome [Title] OR Perimenopausal Syndrome [Title] OR Menopausal Period[Title]) AND (Depression[Title] OR Anxiety[Title] OR Emotional Disorders[Title] OR Depressive Symptoms[Title]) AND (acupuncture[abstract] OR ear acupuncture[abstract] OR Abdominal Acupuncture [Title] OR Scalp Acupuncture[Abstract] OR Body Acupuncture[Abstract] OR Electroacupuncture[Abstract] OR Plum Flower Acupuncture[Abstract] OR Water Acupuncture[Abstract] OR Floating Acupuncture[Abstract] OR Warm Acupuncture[Abstract] OR Navel Acupuncture[Abstract] OR Thread

	Embedding[Abstract] OR Acupuncture Therapy [Abstract]) AND (Random [All Fields] OR Randomized Control[All Fields] OR Randomized Grouping[All Fields] OR rct[All Fields])
PubMed	(((((Perimenopause[MeSH Terms]) OR (Perimenopause[Title/Abstract])) OR (Menopause[MeSH Terms])) OR (Menopause[Title/Abstract])) OR (climacteric[MeSH Terms])) OR (climacteric[Title/Abstract])) AND (((((((Depression[MeSH Terms]) OR (Depression[Title/Abstract])) OR (Depressions[Title/Abstract])) OR (Depressive Symptoms[Title/Abstract])) OR (Depressive Symptom[Title/Abstract])) OR (Emotional Depression[Title/Abstract])) OR (Emotional Depressions[Title/Abstract]))) AND (((((((((((((((Acupuncture[MeSH Terms]) OR (Acupuncture[Title/Abstract])) OR (Pharmacopuncture[Title/Abstract])) OR (electropuncture[Title/Abstract])) OR (manual acupuncture[Title/Abstract])) OR (Acupuncture, Ear[MeSH Terms])) OR (Acupuncture, Ear[Title/Abstract])) OR (Ear Acupunctures[Title/Abstract])) OR (Auricular Acupuncture[Title/Abstract])) OR (Ear Acupuncture[Title/Abstract])) OR (Auricular Acupunctures[Title/Abstract])) OR (Acupuncture Points[MeSH Terms])) OR (Acupuncture Points[Title/Abstract])) OR (Acupuncture Point[Title/Abstract])) OR (Acupoints[Title/Abstract])) OR (Acupoint[Title/Abstract]))
Web of Science	#4#3 AND #2 AND #1<br>#3TS=(Acupuncture or Pharmacopuncture or Electropuncture or manual acupuncture or Acupuncture, Ear or Ear Acupunctures or Auricular Acupuncture or Ear Acupuncture or Auricular Acupunctures or Acupuncture Points or Acupuncture Point or Acupoints or Acupoint<br>#2 TS=(Perimenopause or Menopause or climacteric)<br>#1TS=(Depression or Depressions or Depressive Symptoms or Depressive Symptom or Emotional Depression or Emotional Depressions)

Embase	#10 #3 AND #6 AND #9<br>#9 #7 OR #8<br>#8 'pharmacopuncture':ab,ti OR 'electropuncture':ab,ti OR 'manual acupuncture':ab,ti OR 'acupuncture, ear':ab,ti OR 'ear acupunctures':ab,ti OR 'auricular acupuncture':ab,ti OR 'ear acupuncture':ab,ti OR 'auricular acupunctures':ab,ti OR 'acupuncture pointsr':ab,ti<br>#7 acupuncture<br>#6 #4 OR #5<br>#5 'depressions':ab,ti OR 'depressive symptoms':ab,ti 'depressive symptom':ab,ti OR 'emotional depression':ab,ti 'emotional depressions':ab,ti<br>#4 depression<br>#3 #1 OR #2<br>#2 'menopause':ab,ti OR 'climacteric':ab,ti<br>#1 'perimenopause'/exp OR perimenopause
Cochrane library	#6 #3 AND #4 AND #5<br>#5 (Acupuncture):ti,ab,kw OR (Pharmacopuncture):ti,ab,kw OR (Electropuncture):ti,ab,kw OR (manual acupuncture):ti,ab,kw OR (Acupuncture, Ear):ti,ab,kw OR (Ear Acupunctures):ti,ab,kw OR (Auricular cupuncture):ti,ab,kw OR (Ear Acupuncture):ti,ab,kw OR (Auricular Acupunctures):ti,ab,kw OR (Acupuncture Points):ti,ab,kw OR (Acupuncture Point):ti,ab,kw OR (Acupoints):ti,ab,kw OR (Acupoint):ti,ab,kw<br>#4 (Depression):ti,ab,kw OR (Depressions):ti,ab,kw OR (Depressive Symptoms):ti,ab,kw OR (Depressive Symptom):ti,ab,kw OR (Emotional Depression):ti,ab,kw OR (Emotional epressions):ti,ab,kw<br>#3 #1 OR #2<br>#2 (Menopause):ti,ab,kw OR (Climacteric):ti,ab,kw<br>#1 MeSH descriptor:[Perimenopause] explode all trees
