# Supplementary material for: Acupuncture-adjuvant therapies for treating perimenopausal depression: A network meta-analysis
Source: Medicine (Baltimore). 2023 Aug 18;102(33):e34694. doi: 10.1097/MD.0000000000034694 (PMC10443772; doi:10.1097/MD.0000000000034694)

Supplementary Figure S7.evidence network diagram of KMI score

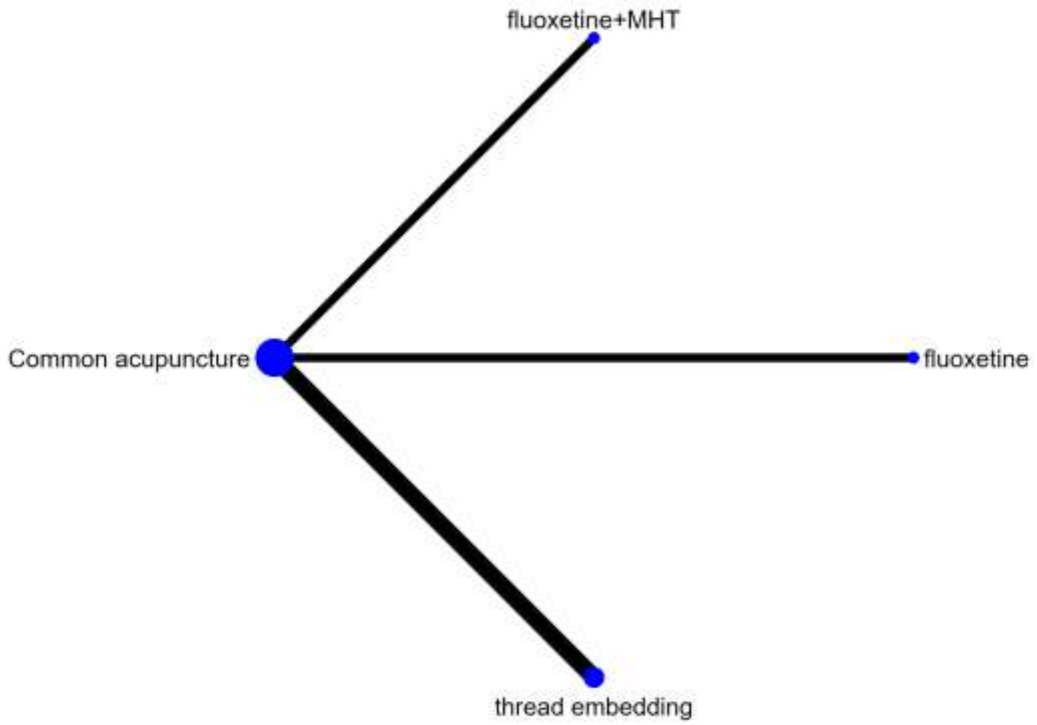

Supplementary Figure S8.evidence network diagram of LH

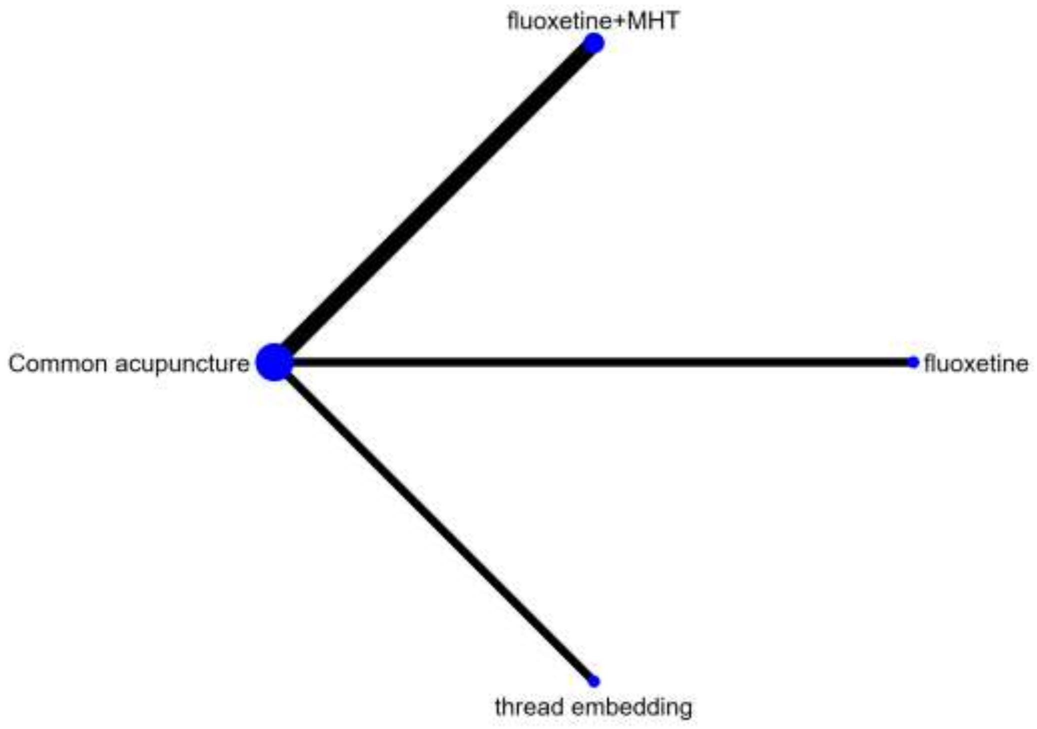

Supplementary Figure S9. evidence network diagram of FSH

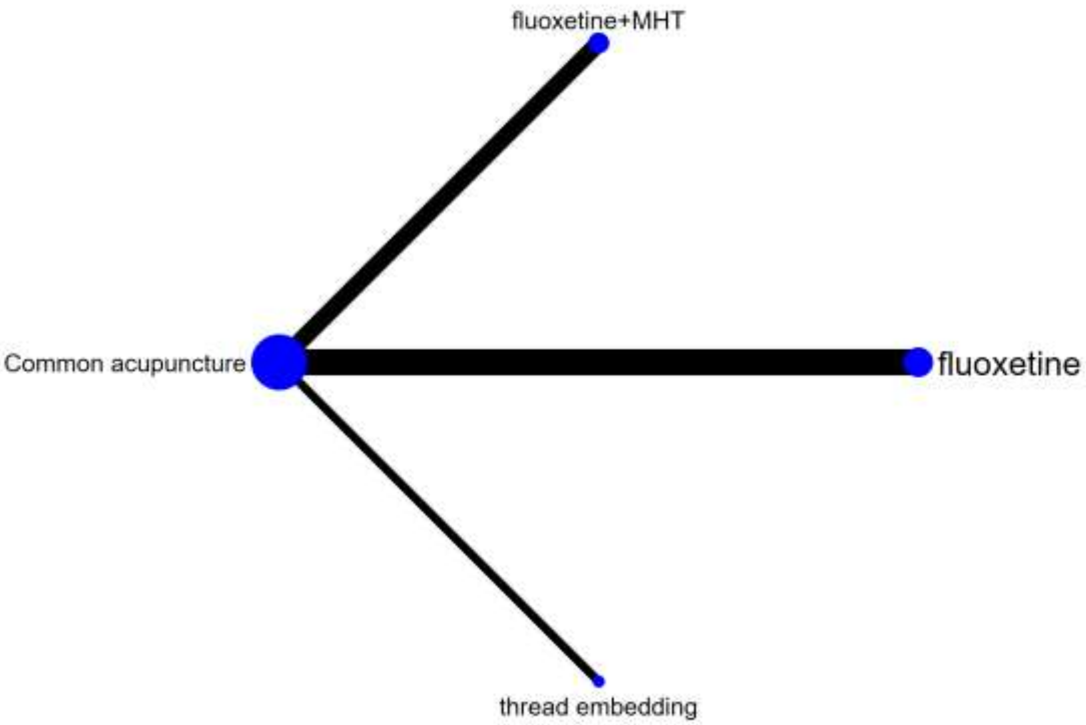

Supplementary Figure S10. evidence network diagram of E2

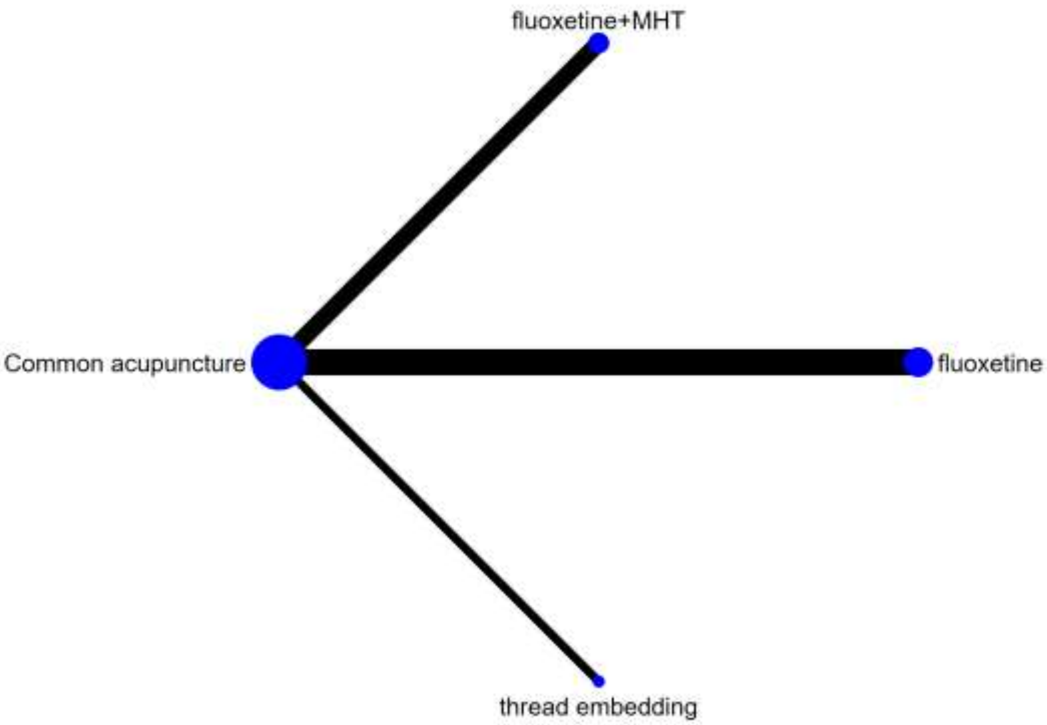

Supplement: Supplementary file 2 [file medi-102-e34694-s002.pdf]
