# Supplementary material for: Acupuncture-adjuvant therapies for treating perimenopausal depression: A network meta-analysis
Source: Medicine (Baltimore). 2023 Aug 18;102(33):e34694. doi: 10.1097/MD.0000000000034694 (PMC10443772; doi:10.1097/MD.0000000000034694)
Supplement: Supplementary file 3 [file medi-102-e34694-s003.pdf]

Table S2. Network analysis of KMI score

Common acupuncture	−0. 03 （−0. 62, 0. 57）	0. 14 （−0. 39, 0. 67）	0. 36 （−0. 29, 1. 01）
0. 03 （−0. 57, 0. 62）	fluoxetine+MHT	0. 17 （−0. 63, 0. 96）	0. 39 （−0. 50, 1. 27）
−0. 14 （−0. 67, 0. 39）	−0. 17 （−0. 96, 0. 63）	thread embedding	0. 22 （−0. 62, 1. 06）
−0. 36 （−1. 01, 0. 29）	−0. 39 （−1. 27, 0. 50）	−0. 22 （−1. 06, 0. 62）	fluoxetine

Table S3. Network Meta analysis of LH levels

Common acupuncture	0. 01 （−0. 41, 0. 44）	0. 16 （−0. 28, 0. 60）	0. 18 （−0. 04, 0. 40）
−0. 01 （−0. 44, 0. 41）	thread embedding	0. 14 （−0. 47, 0. 75）	0. 17 （−0. 31, 0. 65）
−0. 16 （−0. 60, 0. 28）	−0. 14 （−0. 75, 0. 47）	fluoxetine	0. 02 （−0. 47, 0. 52）
−0. 18 （−0. 40, 0. 04）	−0. 17 （−0. 65, 0. 31）	−0. 02 （−0. 52, 0. 47）	fluoxetine+MHT

Table S4. Network analysis of FSH levels

fluoxetine	0. 06 （−0. 46, 0. 57）	0. 12 （−0. 14, 0. 38）	0. 42 （0. 05, 0. 78）
−0. 06 （−0. 57, 0. 46）	thread embedding	0. 06 （−0. 38, 0. 51）	0. 36 （−0. 15, 0. 88）
−0. 12 （−0. 38, 0. 14）	−0. 06 （−0. 51, 0. 38）	Common acupuncture	0. 30 （0. 04, 0. 55）
−0. 42 （−0. 78, 0. 02）	−0. 36 （−0. 88, 0. 15）	−0. 30 （−0. 55, 0. 09）	fluoxetine+MHT

Table S5. E_2 levels network Meta analysis

fluoxetine+MHT	−0. 76 （−1. 55, 0. 03）	−0. 73 （−2. 13, 0. 66）	−1. 60 （−2. 64, −0. 57）
0. 76 （−0. 03, 1. 55）	Common acupuncture	0. 02 （−1. 12, 1. 17）	−0. 85 （−1. 51, −0. 18）
0. 73 （−0. 66, 2. 13）	−0. 02 （−1. 17, 1. 12）	thread embedding	−0. 87 （−2. 20, 0. 46）
**1. 60 （0. 57, 2. 64）**	**0. 85 （0. 18, 1. 51）**	0. 87 （−0. 46, 2. 20）	fluoxetine
