# Supplementary material for: Acupuncture-adjuvant therapies for treating perimenopausal depression: A network meta-analysis
Source: Medicine (Baltimore). 2023 Aug 18;102(33):e34694. doi: 10.1097/MD.0000000000034694 (PMC10443772; doi:10.1097/MD.0000000000034694)

Supplementary Figure S11.SUCRA chart of KMI score

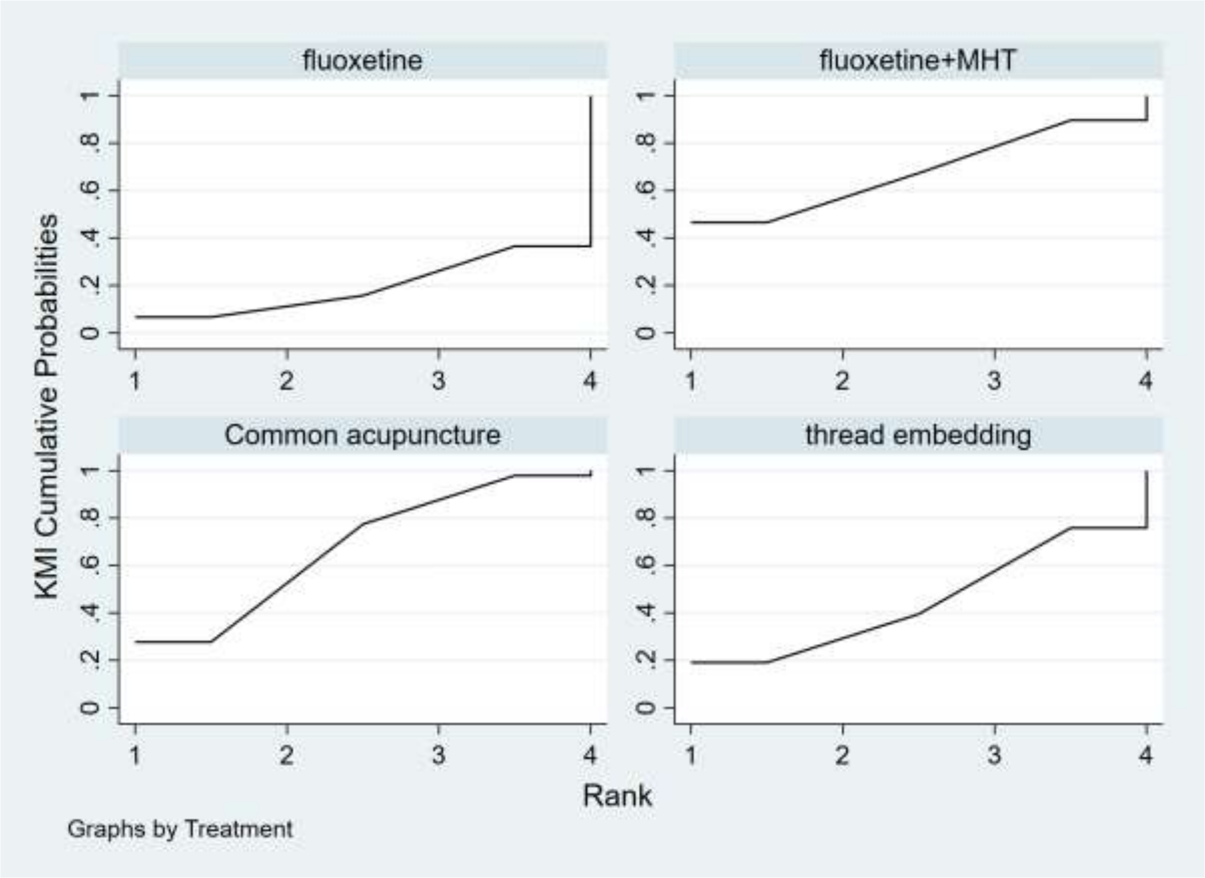

Supplementary Figure S12.SUCRA chart of LH

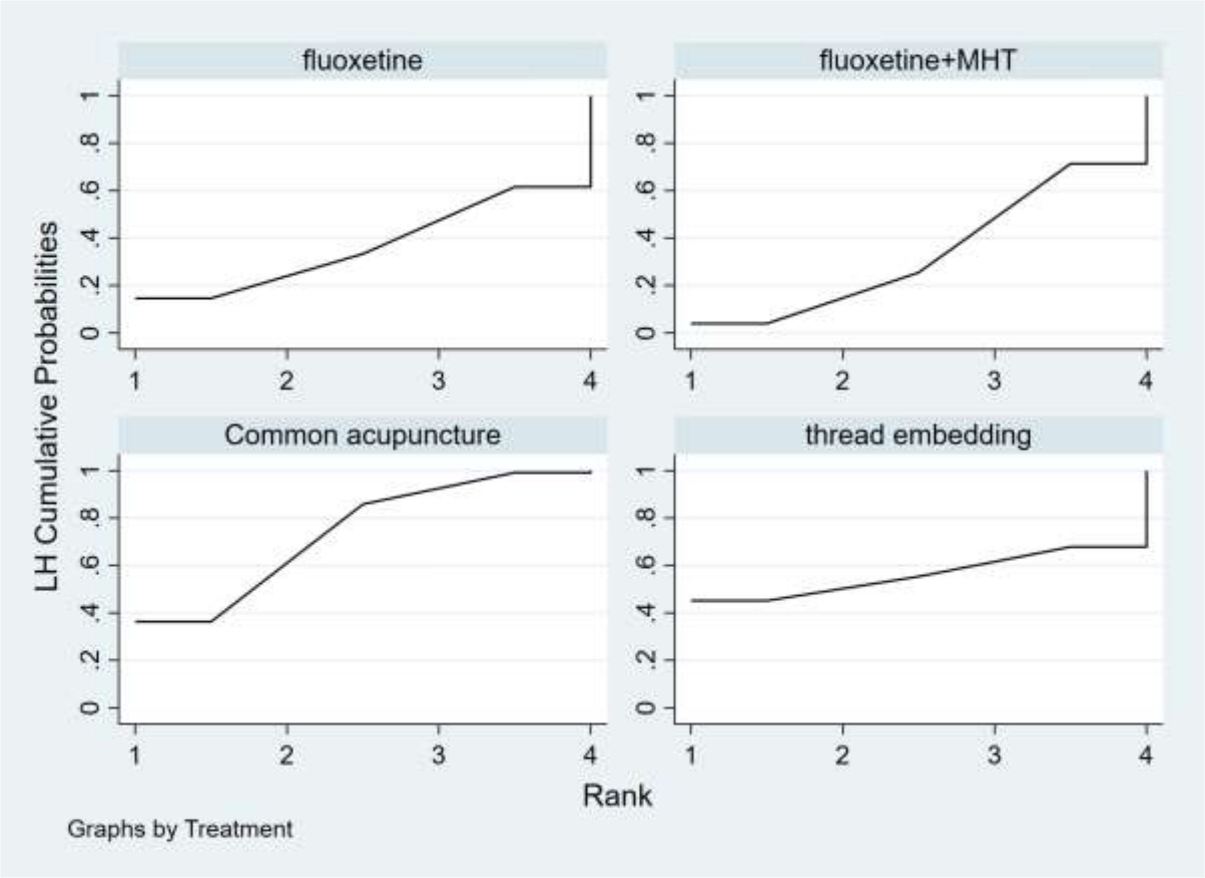

Supplementary Figure S13. SUCRA chart of FSH

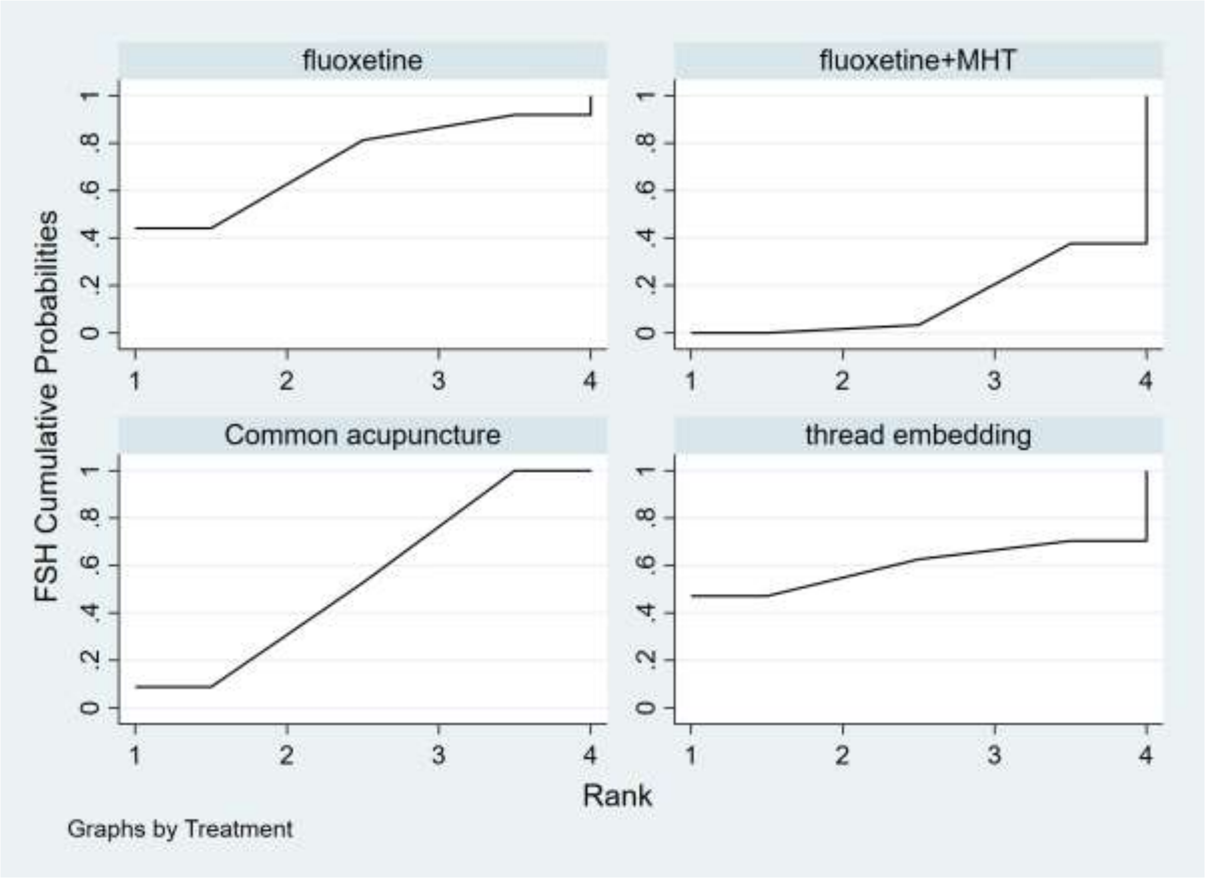

Supplementary Figure S14. SUCRA chart of E2

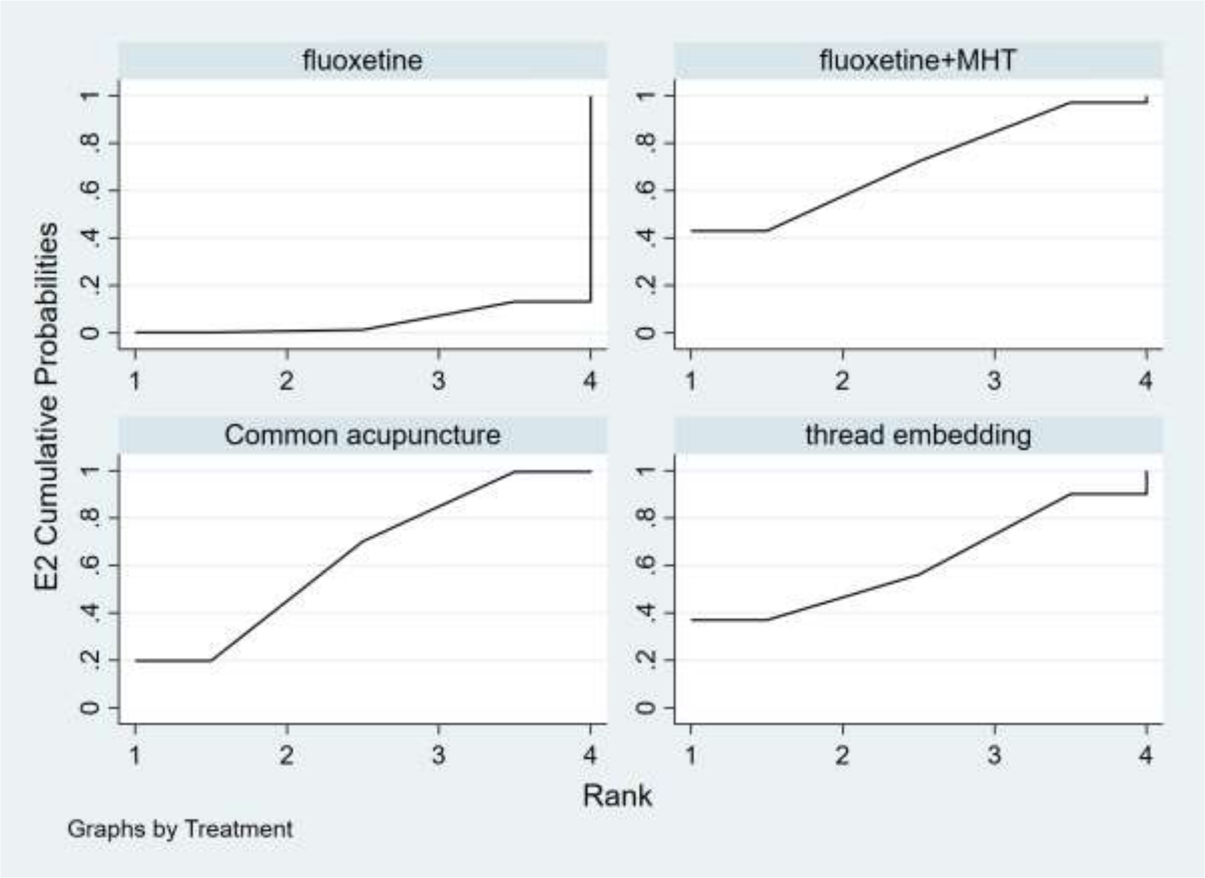

Supplement: Supplementary file 4 [file medi-102-e34694-s004.pdf]
