# Supplementary material for: Acupuncture-adjuvant therapies for treating perimenopausal depression: A network meta-analysis
Source: Medicine (Baltimore). 2023 Aug 18;102(33):e34694. doi: 10.1097/MD.0000000000034694 (PMC10443772; doi:10.1097/MD.0000000000034694)
Supplement: Supplementary file 5 [file medi-102-e34694-s005.pdf]

Results of secondary indicators

KMI score

Evidence Network

5 RCTs reported on KMI scores involving four treatment methods. The overall network relationship was centered on acupuncture. The size of the dots represents the sample size of the intervention, and the thickness of the lines represents the number of RCTs using two-point treatment. See Supplementary Figure S7.

Network meta-analysis

The included studies were subjected to a network meta-analysis, resulting in 6 pairwise comparisons, combined with OR and 95% CI. The network meta-analysis results showed that there was no statistical significance in the comparison between the groups, as shown in Supplementary Table S2 .

SUCRA Probability Ranking

According to the results of SUCRA, warm acupuncture may be the most effective intervention. The results of SUCRA probability from high to low are as follows: fluoxetine> embedding>fluoxetine+MHT> ordinary acupuncture, as shown in the Supplementary Figure S11.

LH score

Evidence Network

4 RCTs reported on LH scores, involving four treatment methods. The overall network relationship was centered on acupuncture. The size of the dots represents the sample size of the intervention, and the thickness of the lines represents the number of RCTs using two-point treatment. as shown in the Supplementary Figure S8.

Network meta-analysis

The included studies were subjected to a reticulated meta analysis, and 4 pairwise comparisons were generated, combined with OR and 95% CI. The results of the reticulated meta analysis showed that there was no statistical significance in the comparison between the groups, as shown in Supplementary Table S3.

SUCRA Probability Ranking

According to the results of SUCRA, warm acupuncture may be the most effective intervention. The results of SUCRA probability ranking from high to low are as follows: ordinary acupuncture> embedding> fluoxetine> fluoxetine + MHT, as shown in the Supplementary Figure S12.

FSH score

Evidence Network

4 RCTs reported on FSH scores, involving four treatment methods. The overall network relationship is centered on acupuncture. The size of the dot represents the sample size of the intervention, and the thickness of the line represents the number of RCTs using two-point treatment. As shown in Supplementary Figure S9.

Network meta-analysis

A network meta-analysis of the included studies was performed, and 6 pairwise comparisons were made, combined with OR and 95% CI. The results of network meta-analysis showed that compared with fluoxetine + MHT, the effect of ordinary acupuncture intervention was better; the other groups are not statistically significant, as shown in Supplementary Table S4.

SUCRA Probability Ranking

According to the results of SUCRA, warm acupuncture may be the most effective intervention. The ranking results of SUCRA probability from high to low are as follows: fluoxetine> thread embedding> ordinary acupuncture> fluoxetine + MHT, as shown in Supplementary Figure S13.

E_2 level

Evidence Network

6 RCTs reported on FSH scores involving four treatment methods. The overall network relationship was centered on acupuncture. The size of the dots represents the sample size of the intervention, and the thickness of the lines represents the number of RCTs using two-point treatment. As shown in Supplementary Figure S10.

Network meta-analysis

From the matrix (Supplementary Table 5), According to the matrix ba,fluoxetine+MHT [SMD1.6,95%CI(0.57 to 2.64)] and Common acupuncture [SMD0.85,95%CI(0.18 to 1.51)] was significantly compared with fluoxetine, and fluoxetine+MHT was significantly inferior to Common acupuncture[SMD1.3,95%CI(0.04 to 0.55)] Supplementary Table S5.

SUCRA Probability Ranking

From the network meta-analysis,fluoxetine+MHT had the highest probability rank (SUCRA=94.2%) in terms of improving E2 level. As shown in Supplementary Figure S14.
