# Supplementary material for: Acupuncture-adjuvant therapies for treating perimenopausal depression: A network meta-analysis
Source: Medicine (Baltimore). 2023 Aug 18;102(33):e34694. doi: 10.1097/MD.0000000000034694 (PMC10443772; doi:10.1097/MD.0000000000034694)

Supplementary Figure S1. An efficient funnel chart

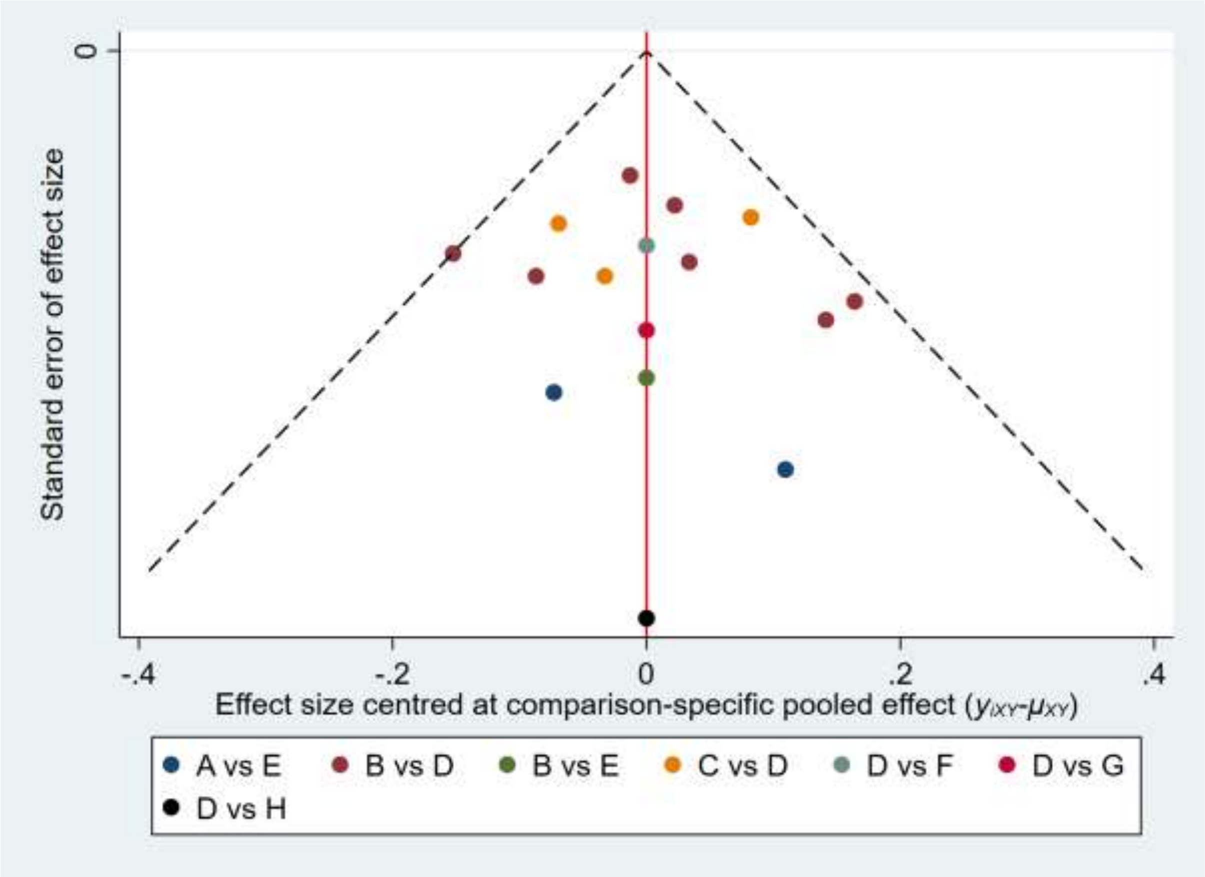

Supplementary Figure S2. Funnel chart of HAMD score

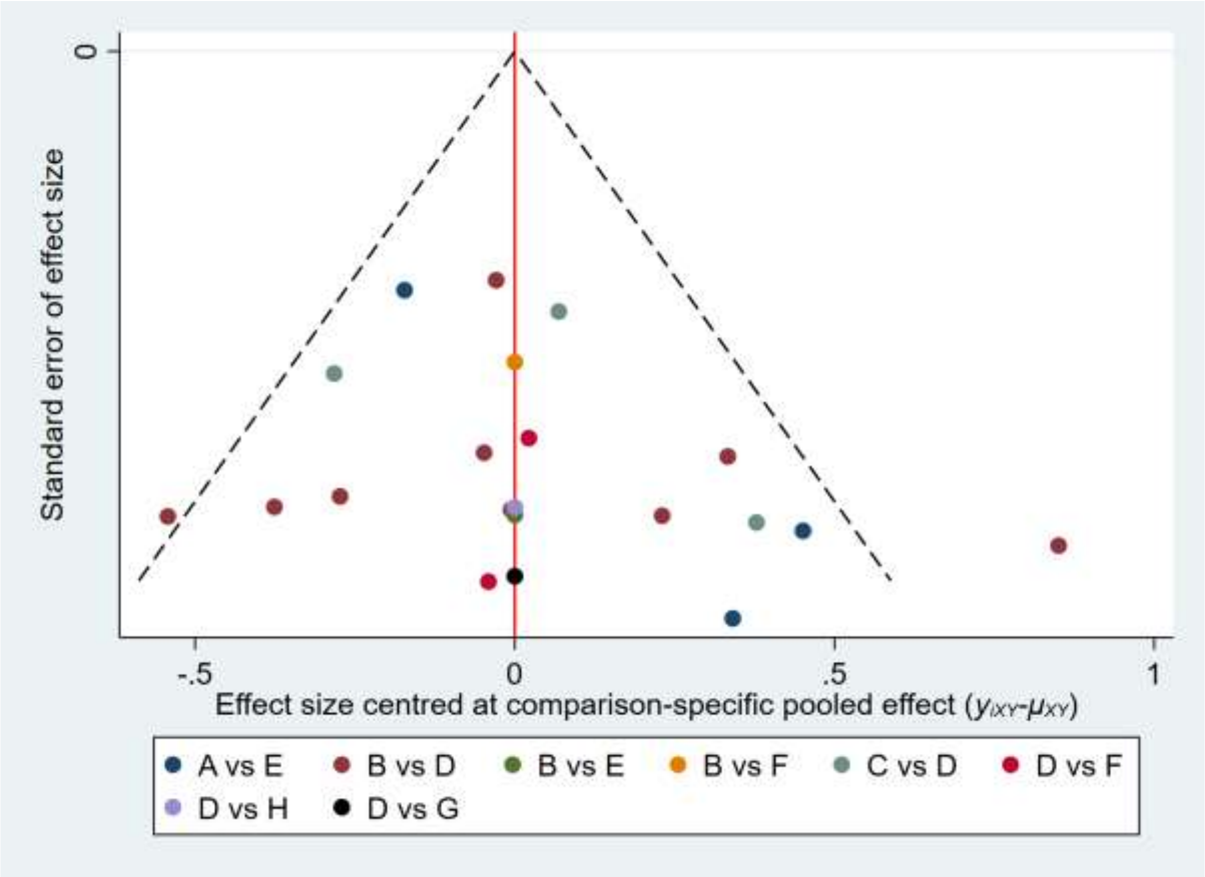

Supplementary Figure S3.Funnel chart of KMI score

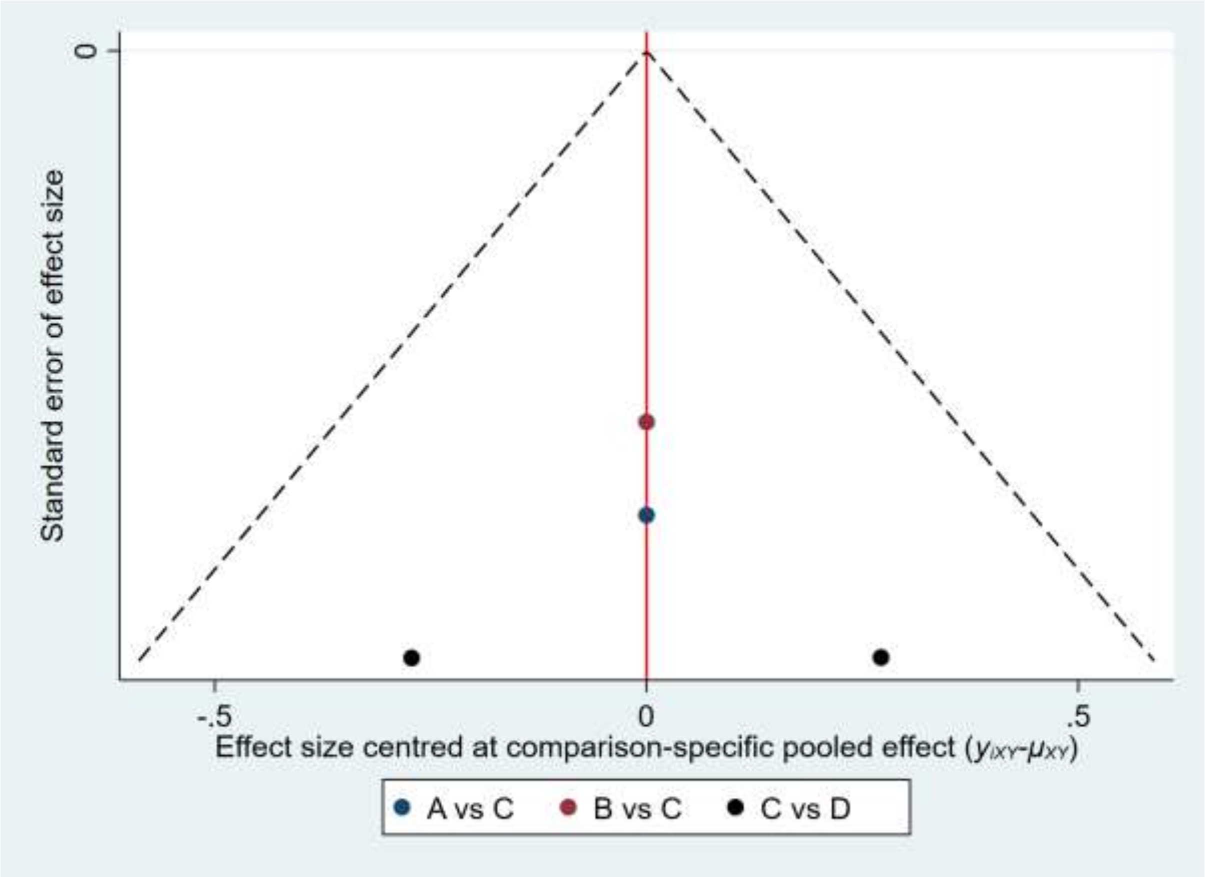

Supplementary Figure S4.Funnel chart of LH

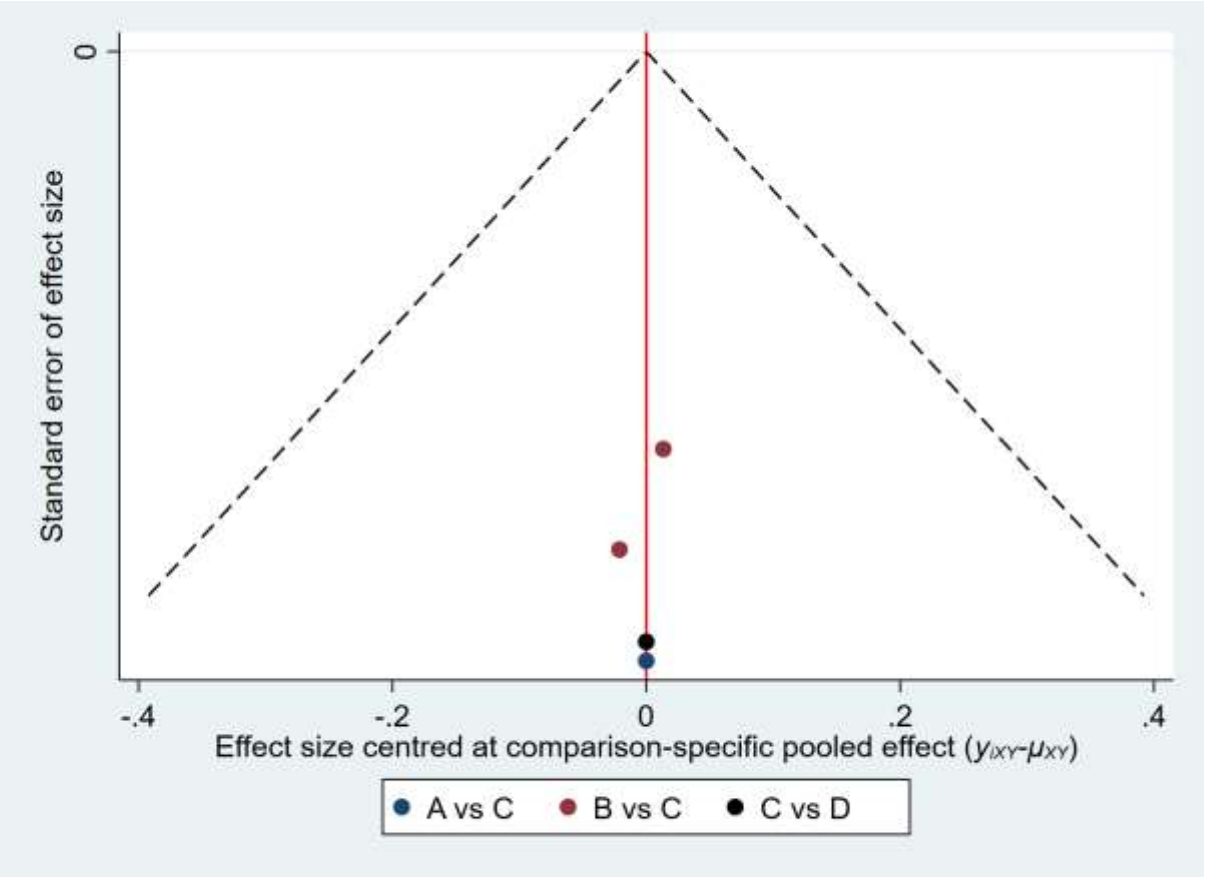

Supplementary Figure S5.Funnel chart of FSH

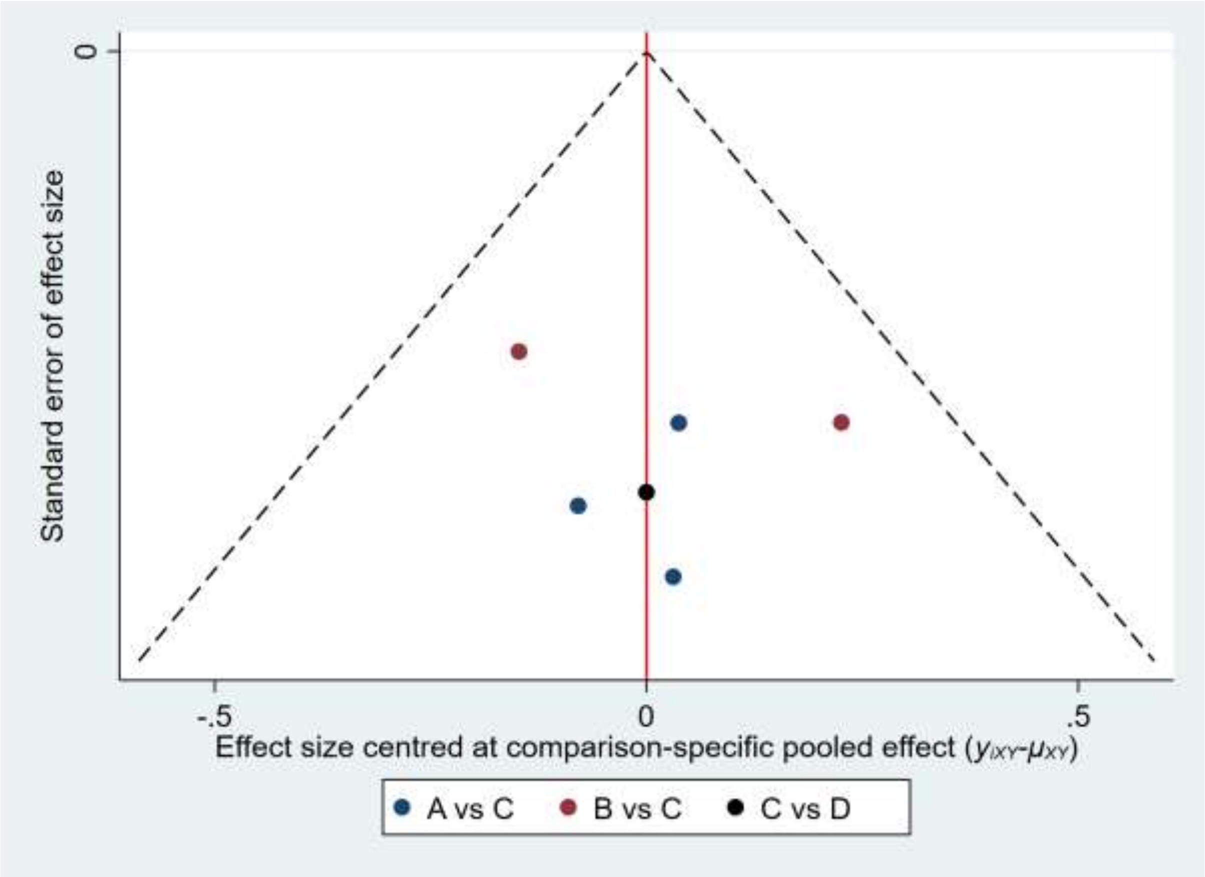

Supplementary Figure S6. Funnel chart of E2

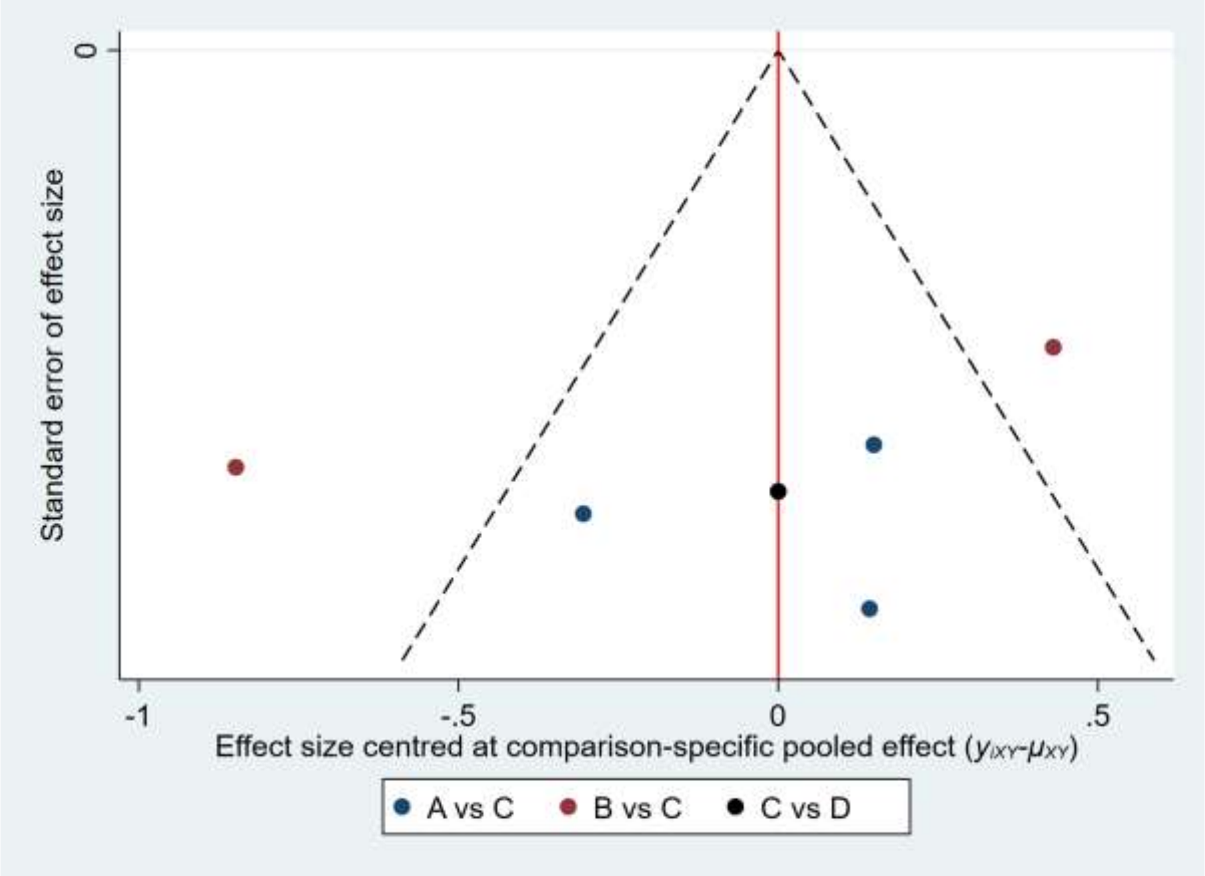

Supplement: Supplementary file 6 [file medi-102-e34694-s006.pdf]
